# Genetic architecture of repeated phenotypic divergence in *Littorina saxatilis* ecotype evolution

**DOI:** 10.1111/evo.14602

**Published:** 2022-09-01

**Authors:** Eva L. Koch, Mark Ravinet, Anja M. Westram, Kerstin Johannesson, Roger K. Butlin

**Affiliations:** ^1^ School of Biosciences University of Sheffield Sheffield UK; ^2^ Department of Zoology University of Cambridge Cambridge UK; ^3^ School of Life Sciences University of Nottingham Nottingham UK; ^4^ Institute of Science and Technology Austria (ISTA) Klosterneuburg Austria; ^5^ Faculty of Biosciences and Aquaculture Nord University Bodø Norway; ^6^ Marine Science, Tjärnö Marine Laboratory University of Gothenburg Gothenburg Sweden

**Keywords:** Divergence with gene flow, local adaptation, structural variants

## Abstract

Chromosomal inversions have been shown to play a major role in a local adaptation by suppressing recombination between alternative arrangements and maintaining beneficial allele combinations. However, so far, their importance relative to the remaining genome remains largely unknown. Understanding the genetic architecture of adaptation requires better estimates of how loci of different effect sizes contribute to phenotypic variation. Here, we used three Swedish islands where the marine snail *Littorina saxatilis* has repeatedly evolved into two distinct ecotypes along a habitat transition. We estimated the contribution of inversion polymorphisms to phenotypic divergence while controlling for polygenic effects in the remaining genome using a quantitative genetics framework. We confirmed the importance of inversions but showed that contributions of loci outside inversions are of similar magnitude, with variable proportions dependent on the trait and the population. Some inversions showed consistent effects across all sites, whereas others exhibited site‐specific effects, indicating that the genomic basis for replicated phenotypic divergence is only partly shared. The contributions of sexual dimorphism as well as environmental factors to phenotypic variation were significant but minor compared to inversions and polygenic background. Overall, this integrated approach provides insight into the multiple mechanisms contributing to parallel phenotypic divergence.

How populations are able to adapt locally and how divergent selection may ultimately lead to speciation (Hereford 2009; Savolainen et al. 2013) have been long‐standing questions in evolutionary biology. Understanding divergence becomes even more of a challenge if local adaptation occurs on very small geographical scales and differently adapted populations remain connected by gene flow (Lenormand 2002). It has been predicted that certain genetic architectures, large effect loci (Yeaman & Otto 2011; Yeaman & Whitlock 2011) or a clustering of locally adaptive alleles with tight linkage between them (Bürger & Akerman 2011; Yeaman and Whitlock 2011; Rafajlović et al. 2016; Aeschbacher et al. 2017), can facilitate local adaptation and make it more resistant to gene flow. If divergent selection is multivariate and acting on multiple traits simultaneously, reduced recombination between locally adaptive alleles contributing to variation in these traits is beneficial (Smadja and Butlin 2011).

Chromosomal inversions can play a major role in the process of local adaptation (Kirkpatrick and Barton 2006). A growing number of studies in a wide range of organisms have demonstrated the importance of inversion polymorphism for local adaptation and divergence (Wellenreuther and Bernatchez 2018). It has often been shown that differently adapted populations differ in the frequencies of alternative arrangements (Jones et al. 2012; Twyford and Friedman 2015; Hanson et al. 2017; Christmas et al. 2019), sometimes with clinal change between populations (Ayala et al. 2014; Kapun et al. 2016; Mérot et al. 2018). The exact mechanisms responsible for the large effects of chromosomal inversions on phenotypes can be diverse. Inversions can disrupt genes at the breakpoints or alter gene expression (Fuller et al. 2016; Lavington and Kern 2017; Huang et al. 2018; Said et al. 2018). In the context of divergent selection with gene flow, where reduced recombination may be beneficial (see above), inversions may be favored because they suppress recombination in individuals that are heterozygous for alternative arrangements (Faria et al. 2019b; Kirkpatrick 2010; Charlesworth and Barton 2018; Wellenreuther et al. 2019). This allows inversions to maintain locally beneficial allele combinations and protect them from the homogenizing effects of gene flow.

Inversions are undoubtedly important, but their importance may be overestimated as they are also relatively easy to detect. Methods for detecting genetic differentiation are biased toward low recombination regions (Burri 2017; Booker et al. 2020). Linkage disequilibrium between genetic markers and loci involved in trait variation increases statistical power in association analyses leading to a potential bias towards regions of low recombination (Roesti 2018). Methods that aim to detect loci involved in adaptation, either by comparing differentially adapted populations (e.g., F_ST_ outlier scans) or by finding associations with traits under divergent selection (e.g., quantitative trait loci (QTL) mapping or genome‐wide association studies (GWAS)) generally have a limited power and are only able to detect loci of relatively large effects. The genetic basis of highly polygenic traits where phenotypic variation is due to many loci of small effect is less easy to study (Pritchard and Di Rienzo 2010). However, even when individual small‐effect loci cannot be identified, it is possible to estimate their joint contribution, the additive genetic variance, using well‐established quantitative genetics frameworks (Falconer & MacKay 1996). A full understanding of the genetic architecture of local adaptation under gene flow necessarily requires a focus not only on a few large‐effect loci (like inversions) but also consideration of the full distribution of effect sizes. Consequently, analyzing the relative importance of inversions requires a quantification of how much they contribute to observed variation when accounting for the contribution of the remaining genetic background outside of inversions.

Another component of variation that should also be considered when assessing the contribution of inversions to local adaptation is a plastic response to different environments. Locally adapted populations, such as ecotypes, are often characterized by morphological differences that might be assumed to have a genetic basis. However, another possibility is that populations are able to change their phenotypes in response to environmental cues (Kawecki and Ebert 2004; Miner et al. 2005; Pfennig et al. 2010). Although studying natural populations in the field, and in the presence of selection pressures that have shaped them, gives us crucial insights, disentangling the effects of genetics and the environment on phenotypic variation along an environmental gradient can be challenging. Contact zones between contrasting habitats where adaptive divergence has evolved provide a great opportunity to study the process and underlying mechanisms for local adaptation for several reasons. They allow us to disentangle environmental and genetic effects by providing us with new combinations of genotypes and environmental conditions. When locally adapted genetically divergent groups come into contact, we can observe individuals with different genetic backgrounds in similar environmental conditions. This is particularly the case for species with a very limited dispersal capability where we can be sure that they grew up close to the sampling location. We may also explore how phenotypes of one ecotype change when individuals migrate into a different habitat and thus, we can estimate how much of the observed phenotypic divergence between ecotypes is due to plastic responses to different habitats. Furthermore, contact zones allow us to get insights into the mechanisms that keep locally adapted groups distinct even when they are within dispersal range of each other and could hybridize. Gene flow can be reduced on small spatial scales by various mechanisms including different habitat preferences, assortative mating, genetic incompatibilities, and selection against hybrids or migrants. All of these are impossible to investigate if only geographically clearly separated groups are studied. If hybridization occurs, it provides us with genetically admixed individuals that can be used to pinpoint specific genomic regions associated with phenotypic variation.

In this study, we investigated the complex interplay of different factors responsible for phenotypic variation in the marine snail *Littorina saxatilis*. This species has evolved an amazing phenotypic diversity (Reid 1996). Two relatively well‐studied ecotypes are associated with different parts of the shore (Johannesson et al. 1993; Panova et al. 2006; Butlin et al. 2014; Johannesson 2016). The “Wave” ecotype inhabits wave‐exposed rocks and is characterized by a small size, a thin shell with a globular shape, and a relatively large aperture (see also Fig. 1a). These features help snails to stay attached to the rock surface and prevent dislodgement by wave action (Le Pennec et al. 2017). Snails living in boulder fields are more protected from wave action but suffer from crab predation. This “Crab” ecotype is usually much larger, has a more elongated shell with a high spire and a relatively small and narrow aperture that prevents crabs from cracking the shell or pulling out the snail (Johannesson 1986; Boulding et al. 2017). The ecotypes appear to have evolved in situ and repeatedly in several different areas (Butlin et al. 2014) and genetic differentiation between them is low (F_ST_ = 0.04 in Sweden; Westram et al. 2021). Phenotypes change gradually across the Wave‐Crab contact zone from one habitat to the other and there is ongoing gene flow between ecotypes (Johannesson 2016; Westram et al. 2018). A high number of polymorphic chromosomal inversions exists in this species (Faria et al. 2019a; Figure 1c) with some of them showing frequency differences in alternative arrangements and significant clinal patterns across the habitat transition (Westram et al. 2018, Westram et al. 2021).

**Figure 1:**
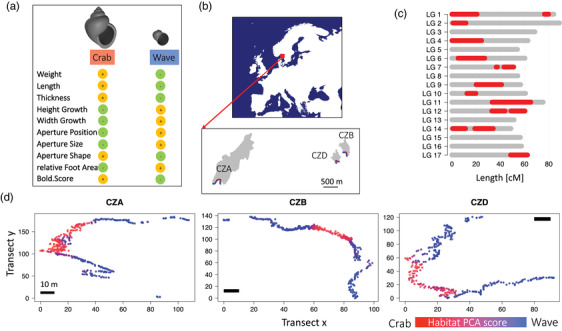
(a) Traits analyzed in this study and their association with ecotypes. Yellow+ indicates that larger values are associated with the respective ecotype, green‐ indicates smaller values. Figure modified from Koch et al. 2021. (b) Location of the sampling sites on three Swedish islands. Distances between islands are 4.5 km between CZA and CZB, 4.1 km between CZA and CZD, and 0.4 km for CZB and CZD. (c) Distribution of inversion regions (red) on linkage groups (LG) (Faria et al. 2019a; Westram et al. 2021). For inversion regions that showed recombination suppression in these studies (on LG1, LG4, LG10), we show length based on Koch et al. (2021) where no recombination suppression occurred, and length of linkage groups and sizes of inversion regions are more representative. (d) Positions of samples at the three sites in two dimensions. Colour refers to habitat PC1 that is based on substrate type (boulder or bedrock), presence/absence of barnacles (indicator of wave exposure), and presence/absence of fucoid seaweed (indicator of more sheltered habitats). Lower habitat PC scores (red) refer to boulder fields where snails are exposed to crab predation and higher habitat PC scores (blue) to wave exposed rocks.

Using lab‐reared F2‐individuals that resulted from crosses between ecotypes for QTL‐mapping, it was shown that loci contributing to phenotypic variation in traits under divergent selection coincided with inversion regions (Koch et al. 2021). However, some of the traits were also found to show adaptive plasticity, being able to respond to both crab cues and wave exposure (Hollander and Butlin 2010); thus the extent to which inversions contribute to phenotypic variation under natural conditions is not clear. Studying individuals along a habitat transition in the presence of the selection pressures under which ecotypes have evolved and the environmental factors that may influence phenotypes can help to study the joint contributions of all these factors. Quantitative genetic methods offer a useful and well‐established framework to estimate the variance due to additive genetic variation. In addition, they allow the incorporation and effect estimation of other factors. Here, we included inversion genotypes as fixed effects while controlling for polygenic additive effects in the collinear genomic background, enabling us to estimate the effects of inversions in a unified framework. Furthermore, we accounted for environmental factors to which traits may respond via phenotypic plasticity. Applying this approach to data from replicate contact zones allowed us to further explore the consistency of inversion effects and repeatability of the genomic architectures of traits under divergent selection. This provided detailed insights into the genetic mechanisms of phenotypic divergence under gene flow including the contribution of large effect loci (inversions), polygenic effects, and the influence of environmental factors.

## Methods

### SAMPLING AND PHENOTYPING

Samples are from Westram et al. (2021), where inversion and SNP clines in different contact zones were studied. Snails were collected along the Swedish West coast, where Crab and Wave habitats frequently come into contact (Fig. 1b), in 2013 and 2014. The sites were Ramsö (58°49’27.8N11°03’45.3E), here CZA, Inre Arsklovet (58°50’00.5N 11°08’19.6E), here CZB, and Yttre Arsklovet (58°49’51.3N11°07’59.0E), here CZD. At each location snails were collected along a transect that started in the wave habitat (exposed bedrock) continued to the crab habitat (boulder fields in the center of a bay) and extended into the next wave habitat at the other side of the bay (Fig. 1d). Our data set consisted of 1130 individuals (CZA: 379, CZB: 381, CZD: 370). We analyzed data from each island separately. The snails are direct developers with internal fertilization and no dispersing larva stage. Embryos develop inside a brood pouch. This results in a very low dispersal capability. Gene flow between islands is thus very limited and evolution of the populations on each island occurs largely independently. Exact positions of all individuals were recorded in three‐dimensional space using a Total station (Trimble M3). Positions along the transects were then converted to a one‐dimensional distance measurement along a least‐cost path (see Westram et al. 2021) that minimized path length and constrained movement to areas of high snail density. The first snail collected along the transect path (starting in the Wave habitat, Fig. 1d) was assigned position 0. Total path lengths were: 362.47 m (CZA), 257.00 m (CZB), 270.17 m (CZD). Environmental variables were recorded to describe the habitat at 1000–2000 points for each transect. These included substrate type (bedrock/boulder), presence of barnacles as indicator of wave exposure, and presence of fucoid seaweed (indicator of weak wave action and more sheltered habitats). These measurements were then summarized as “habitat PC” by a principal component analysis using the “princomp” function in R (R Core Team 2020).

Sex of each snail was determined by dissection. Weight (wet weight of snails including shell), shell length, and thickness were recorded. Thickness was measured with a thickness gauge (NeoteckDTI Digital Dial Indicator Probe, 0.001mm resolution) at the widest point of the aperture and the average of three measurements per individual was used. Foot area was measured from photos of snails moving inside seawater‐filled Falcon tubes using the program ImageJ (http://imagej.nih.gov/ij/). Foot area (in cm^2^) was divided by shell length to obtain relative foot area. For describing shell shape and aperture characteristics, several size‐independent parameters (height‐growth, width‐growth, aperture‐position, aperture‐size, aperture‐shape) were used, based on a growth model developed by Larsson et al. (2020). They describe whether the shell shape is more elongated with a high spire (small height‐ and width‐growth) or globose (large growth parameters). Small values for aperture‐position and large values for aperture‐shape indicate narrow apertures, wide apertures have a large aperture‐position and small values for aperture‐shape (Fig. 1a). Boldness behavior was measured as Bold Score (details in Koch et al. 2021), which is the log‐time until an individual crawls out of its shell after disturbance (higher Bold Score means individuals are less bold). Each individual was measured three times and the average was used as a boldness proxy.

### GENOTYPING

The genetic data had been used for cline and outlier analysis in Westram et al. (2021). Details for DNA extraction, library preparation, and sequencing can be found there. A targeted capture sequencing approach (paired‐end 125 bp sequencing) was applied for genotyping using 40,000 probes of 120 bp length. Poorly represented regions were filtered out by only retaining contigs with at least 10 reads in a minimum of 500 individuals across all locations. SNPs were called using samtools (Li et al. 2009) mpileup and bcftools call, including only bases with a quality of at least 20. SNPs were filtered and only biallelic SNPs with a variant quality of at least 20, a minor allele frequency of at least 0.1, and at least 150 individuals with data from each site were retained. Inversion genotypes are based on Westram et al. (2021). Additionally, we genotyped individuals for inversions on linkage group (LG) 12 that had not been determined before. We applied a PCA on all markers within the previously described inversion regions (Faria et al. 2019a) and two additional putative inversion regions (Hearn et al. 2022) using the “prcomp” function in R. We detected three main clusters of individuals for two regions on LG 12 as expected for a polymorphic inversion with two alternative arrangements and grouped them into three groups (using the “kmeans” function) reflecting the two homozygous and the heterozygous genotypes (Fig. S1). Some of the inversions are complex (on LG6 and LG14) because of more than one inversion event within the same region (Faria et al. 2019a), resulting in three arrangements segregating in the population. However, one of the haplotypes was consistently at low frequency and did not show a clinal pattern along the habitat transition. Most individuals were thus homozygous or heterozygous for the two common arrangements. We therefore used only genotypes of one of these arrangements (i.e., number of copies per individual). The resulting data set included one genotype for each individual and inversion.

### STATISTICAL METHODS

Statistical analyses were performed in R version 4.0.3 (R Core Team 2020).

We calculated an “inversion free” pairwise genomic relationship matrix (GRM) for each site separately using only SNPs outside the inversion regions with 5 cM buffer around them. For this, we used the previously published linkage map (Westram et al. 2018) and positions of known inversions based on Faria et al. (2019a) and Westram et al. (2021). We used the methods proposed by Yang et al. 2010, Yang et al. 2011) as included in the Rpackage “AGHmatrix”(Amadeu et al. 2016). Based on these GRMs, median relationships of each snail to all other individuals within certain distances along the sampling path (1, 2, 3, 5, 10 m) were calculated.

We fitted quantitative genetic linear mixed models (animal models) (Kruuk 2004; Wilson et al. 2010) with each phenotypic trait as response variable. These models can estimate the variance due to additive genetic effects (V_A_) by using information on pairwise relationships between individuals as random effect and allowing the incorporation of fixed effects. Additive genetic effects are included as random effects with a covariance structure determined by a pairwise relatedness matrix.

The model is:

y=Xβ+Zu+e

where *y* is a vector of observed phenotypes, β are fixed effects with X being the design matrix that links fixed effects to the corresponding individuals. Z is a design matrix relating the random effects, here the additive genetic effects *u*, to individuals and *e* is a vector of errors. V(*u*) = A σ^2^_A_ where A is the pairwise relationship matrix and σ^2^_A_ is the additive genetic variance. Instead of using a relationship matrix based on a pedigree we used estimated relationships (pairwise genomic relationships GRMs) based on genomic markers outside inversion regions as described above. All models were run in ASReml‐4 (VSN International, Hemel Hempstead) implemented in Asreml‐R (Butler et al. 2017; Butler 2021). Models were fitted to variance and mean standardized trait values (*z*‐scores). This standardization enables comparisons between the different traits and gives estimates of the narrow sense heritability directly. As fixed effects, we included habitat (habitat PC), shore height, and the distance from the center of the crab habitat (see Westram et al. 2021 for definition of habitat boundaries) to account for potential isolation by distance effects that could occur independently of the habitat transition. The inversion genotypes of 16 inversions (coded as 0 and 2 for homozygotes and 1 for heterozygous) and sex of each individual were also included as fixed effects. We transformed variables to *z*‐scores to make effects of these different factors comparable. Missing values were replaced with zeros (mean of standardized values). Significance of fixed effects was assessed by conditional Wald tests as implemented in ASReml. We corrected for testing significance of each fixed effect on multiple traits using the false discovery rate (FDR). For testing significance of V_A_ (additive genetic variance outside inversion regions), we compared the full model to a model without additive genetic effects using a likelihood ratio test with one degree of freedom. We also applied models including interactions between habitat, shore height, and sex. We used variance inflation factors (VIF) to check for collinearity of our variables and found most of them to be low (< 2). Habitat PC (3.2‐4.4), distance from center (2.7–4.0), and inversion 14.1 (2.0‐3.5) showed moderate VIF. All models reached convergence except those for shell thickness that failed at CZA and CZD and were therefore excluded.

After running the linear mixed models that included sex, environmental variables (habitat, shore height), distance from center of the crab habitat, inversion genotypes as fixed effects, and additive genetic effects (based on genomic markers outside inversion regions, see above) as random effects, we partitioned the observed phenotypic variation into different components to calculate proportions of variance that can be attributed to different factors. V_A_ was retrieved from model output of Asreml. For calculating variance explained by fixed effects, we followed de Villemereuil et al. (2018). To give variance explained by inversions we combined the effects of all inversions, which were included as separate fixed effects in the model.

## Results

**Figure 2:**
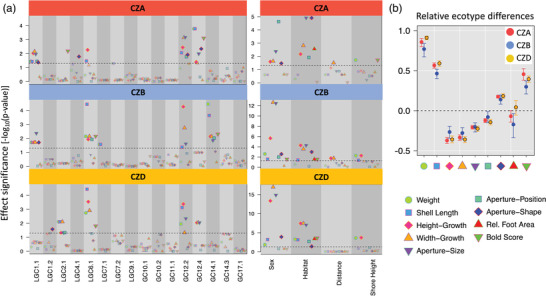
(a) Significance of inversion (left panel) and other effects (right panel) on trait variation. Shown are −log_10_ of *p*‐values on the *y*‐axis. Significance was assessed by using conditional Wald‐tests. *p*‐Values were adjusted for testing multiple traits using the false discovery rate (FDR). The dashed line indicates the significance threshold (FDR = 0.05). (b) Relative differences between ecotypes including 95% Confidence intervals. Only individuals sampled in the defined Crab and Wave habitat were included. The relative differences were calculated as the difference between average Crab and Wave individuals divided by average Crab phenotype. Confidence intervals are based on bootstrapping (10,000 iterations).

The measured traits showed consistent changes across the transect, i.e., the direction of change in the Crab habitat was the same at all sites (Fig. 2b and Fig. S3). Differences between ecotypes (relative to average Crab ecotype) were similar with slightly smaller differences at CZB (Fig. 2b)

### Inversion effects

We found that most traits were significantly affected by inversion polymorphisms. The significance of inversion effects varied between traits as well as between sites. Inversions with the most consistent effects were LGC6.1 and LGC12.2. LGC6.1 showed a significant effect on shell length and the shell shape parameter height‐growth at all sites (Fig. 2a, left panel), whereas LGC12.2 consistently influenced shell length, shell shape (height‐growth and width‐growth), and aperture‐size. Some inversions showed significant effects at only two of the sites: LGC1.1 at CZA and CZB on size (shell length and weight), shell shape and aperture‐size and aperture‐shape, and LGC12.4 on height‐growth and width‐growth as well as aperture‐size at CZA and CZD. In a few cases, inversions showed effects at only one single site. At CZB, LGC14.1 had a strong influence on several traits whereas it was not significant at any other site. We generally found that significant inversions influenced more than one trait and often showed simultaneous effects on size and shell shape. Although phenotypic divergence is very consistent among islands, the underlying genetic mechanisms, here the contribution of specific inversions, is only partly shared between sites.

### Other effects

At all sites, we found significant sexual dimorphism for the shell shape parameters height‐growth and width‐growth as well as aperture‐size (Fig. 2a, right panel, see also Fig. S4, Table S1), evident by a significant sex effect in the models. The habitat PC that we included in the models summarizes substrate type and wave exposure (indicated by presence of barnacles and absence of seaweed). It had a significant influence on almost all traits suggesting that they show some degree of plasticity and can change in response to the habitat (substrate and exposure) independently of genetic changes. This was particularly the case for shape‐ and aperture‐related parameters, most of all height‐growth and width‐growth and aperture size (Fig. 2). Shore height showed a significant effect on weight at all three sites and was significant for height‐growth at CZB and CZD (Fig. 2). Distance from the center of the crab habitat, which we included to account for potential isolation by distance effects or changes along the transect not included in the habitat PC, showed no significant effect in most cases with the exceptions of height‐growth and width‐growth at CZB.

### Interactions

**Figure 3:**
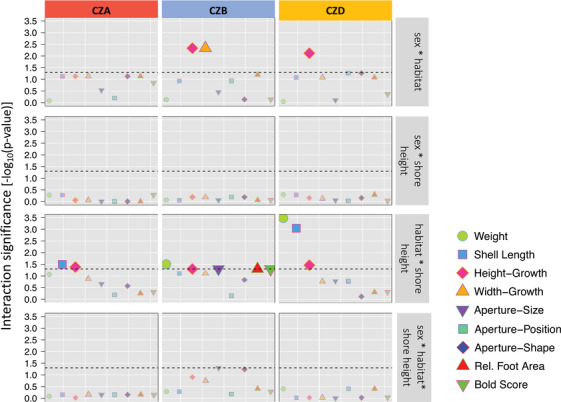
Significance of interactions of different traits at three sites. −log_10_ of *p*‐values (based on Wald tests) are on the *y*‐axis. *p*‐Values were adjusted for testing multiple traits using FDR. The dashed line indicates the significance threshold (FDR = 0.05). Smaller transparent points represent traits without significant interactions.

**Figure 4:**
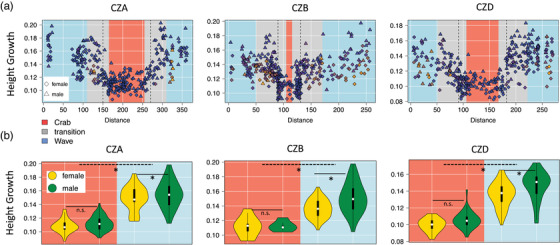
Examples of how some of the traits changed in relation to variables. a: Height Growth along the transect colored by shore height (yellow = upper shore, blue = lower shore). Background colors indicate the habitats: blue = “pure” Wave habitat, red = “pure” Crab habitat, grey = transition zone. Dashed vertical lines indicate the habitat transition. Different distances from the transition for Crab and Wave boundaries account for the differences in dispersal between ecotypes. In addition to the clear difference between Crab and Wave habitat, there was a significant influence of shore height on height growth. For other traits see Fig. S5. (b) Differences between females and males of both ecotypes in height‐growth. Asterisks indicate significant differences, n.s non‐significant. Dashed lines are the between‐ecotype comparisons and solid lines are the comparisons between sexes. Most traits showed significant differences between snails sampled in the Crab and Wave habitat as well as between sexes. At CZB and CZD there was a significant interaction between sex and habitat (see also Fig. 3). Plots for other traits can be found in the Supporting Information (Figure S4).

We tested for interactions between habitat, sex, and shore height. There were interactive effects between shore height and habitat for several traits (Fig. 3), which were consistently significant for height‐growth. As a general pattern, we found that snails higher up the shore in the wave habitat exhibited phenotypes that were more similar to the Crab ecotype (Fig. 4a, see also Fig. S5). There were no interactions between shore height and sex at any of the locations. We found significant interactions between sex and habitat at CZB and CZD for height‐growth and for width‐growth at CZB indicating that the degree of sexual dimorphism changes in different habitats and was higher in the Wave area (Fig. 4b).

### Variance explained

Intensive sampling in the contact zones provided us with individuals from different habitats with different combinations of inversion karyotypes and admixed genomic backgrounds. Small to moderate VIF values indicated limited collinearity thus giving us the possibility to disentangle effects. The reason to include distance in addition to habitat PC despite showing collinearity was to control for potential isolation by distance effects and potential other environmental effects to get an unbiased estimate of the inversion effects. We repeated the analysis without distance from the center and found the results to be the same (Fig. S7).

**Figure 5:**
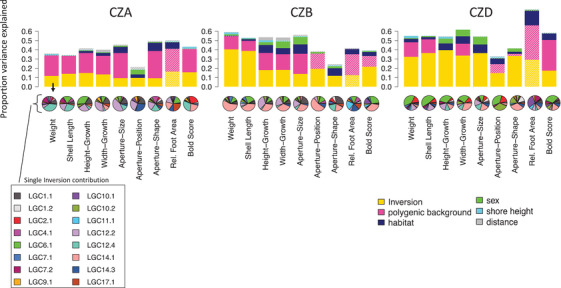
Estimated variance components as proportions of total phenotypic variance. Shaded areas (white diagonal lines) represent non‐significant effects. The yellow bars in the upper panel represent the variance explained by all inversions combined. The contribution of each individual inversion is given in the pie charts below.

At all sites, inversions and polygenic additive genetic effects in the remaining genome (captured by GRMs based on markers outside inversion regions) represented the major parts of the variance explained. There were slight differences between the sites. CZA showed the smallest proportion of variance explained by inversions and the highest proportion of additive genetic variance outside inversions (V_A_) (Fig. 5). Aperture‐related traits (aperture‐position, aperture‐shape at CZB and CZD, aperture‐size at CZD) often showed non‐significant or low amounts of V_A_. Estimates of V_A_ and inversion effects were non‐significant for relative foot area (Fig. 5), which was the only trait without clear changes along the transect (Fig. S3) and no differences between ecotypes (Fig. 2b). This trait was hard to measure, likely resulting in noisy and inaccurate phenotypic values. At CZD, reliable estimates were further impeded by limited sampling in the Wave habitat. Although habitat PC showed very clear changes across the transects, it explained only small to moderate levels of phenotypic variance in the measured traits and considerably less than V_A_ and inversion effects (Fig. 5) indicating a limited contribution of phenotypic plasticity. Sex and shore height had significant effects on several traits (Fig. 2), but they explained only small amounts of the total variance at each site. The highest proportions for sex were mainly found in height‐growth and width‐growth and aperture‐size. Variance explained by distance from the center was very small and, in most cases, non‐significant indicating that most of the influential environmental variation, which changes along the transect, was captured in the habitat PC.

In some cases, we found that factors with significant influences seemed to counteract each other. We observed that values for the shell shape parameter height‐growth predicted by shore‐height and inversions showed a negative covariance, i.e. they diminished each other's effect at CZB and CZD (see also Fig. S6). We found indeed that higher shore position in the Wave habitat counteracts the main ecotype divergence and results in individuals that are more similar to the Crab type (Fig. 4a, Fig. S5).

Variances explained by inversions depend on effect size but also on genotype distribution and frequency at each location. For example, an inversion arrangement with a strong and significant effect on a trait will only negligibly contribute to overall variation if it is very rare. To gain a better understanding of the differences between locations, we compared variation in inversion arrangement frequency (i.e., variance of the inversion genotypes coded as 0, 1, and 2) and found it to be very similar across locations (Fig. S8, see also Fig. S2) indicating that differences in variance explained by inversions are not due to arrangement frequency differences between sites. Next, we compared estimated effects at the three locations (Fig. S10, Table S1). Inversions with significant effects at all sites (LGC6.1 for shell length, height‐growth, LGC12.2 for shell length, height‐growth, and width‐growth) showed very similar effect sizes. Inversions significant at one or two of the locations had usually smaller effects but in the same direction at the non‐significant locations indicating overall consistent effects across the three studied sites.

### Relatedness

**Figure 6:**
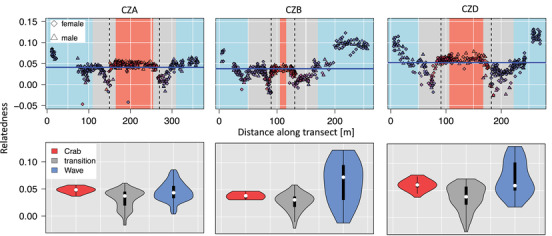
Median relatedness of individuals to others within 10 m distance along the transect. Point colors in the upper panel refer to hybrid indices (based on Johannesson et al. 2019); red = pure Crab, blue = pure Wave. Background colors indicate the habitats: blue = “pure” Wave habitat, red = “pure” Crab habitat, grey = transition zone. Dashed vertical lines indicate the habitat transition. Different distances from the transition for Crab and Wave boundaries account for the differences in dispersal between ecotypes. The blue horizontal line shows the average relatedness within 10 m distance. There is a decrease in relatedness some meters before the habitat transition. This pattern is independent of the distance used for calculating median relatedness (see Fig. S12). The comparison between individuals sampled inside the transition zone and in the Crab/Wave habitats (violin plot in the lower panel) shows that individuals in the transition zone have a lower relatedness to the surrounding individuals.

We calculated median relatedness of each individual to others within 10 m distance along the transect (Fig. 6). In general, we found that closely related individuals were also close in space indicating limited dispersal (Fig. S11). However, median relationships showed clear reductions close to the habitat transitions (Fig. 6). The most extreme reduction in relatedness was found towards the Wave habitat a few meters away from the defined habitat boundary (dashed vertical line in Fig. 6). This pattern did not change when different distances were used for calculating relatedness (Fig. S12). Considering the hybrid indices of individuals around the transition (Fig. 6), it seems that Crab individuals moved into the Wave habitat, which is consistent with the observed displacement of clines into the Wave habitat (Westram et al. 2021).

### Variance partitioning across linkage groups

For most traits, variance partitioning showed a positive relationship between variance explained by additive genetic effects of linkage groups (LGs) and LG length, with several LGs contributing significantly to trait variation (Fig. S13) indicating many loci contributing to the trait. We found that LG 12 contributed disproportionately to observed variance in several traits, consistent with the results of the inversion effect testing that showed significant effects for inversions on this LG. We also detected a disproportionate contribution of LG 6 to shell length. Traits with low or non‐significant V_A_ (aperture‐position, Bold Score, relative foot area) showed lower LG‐specific heritability estimates. Overall, the results are consistent with the other analyses: LGs with inversions of significant effect (e.g., LG 12, LG 6, LG 14) often contribute disproportionately, but a polygenic basis, small additive effects of the remaining genome, is also present as shown by significant contributions of several LGs, including those without known inversions, and the overall positive relationship with LG length.

## DISCUSSION

### Evaluating the contribution of inversions to phenotypic variation

Arrangement frequency differences between differently adapted populations or geographic clines strongly suggest the importance of inversions for local adaptation in a broad range of organisms. This has further been supported by crossing experiments in some systems. The impact of inversions on traits under selection could be shown by using admixed individuals and homogenising genomic backgrounds, e.g., in QTL mapping in *Mimulus guttatus* (Coughlan and Willis 2019), crossing experiments in *Drosophila* (Durmaz et al. 2018) or introgressing inversion arrangements into an alternative genomic backgrounds (Lowry and Willis 2010; Crow et al. 2020). While controlling for genomic background represents a powerful way of confirming inversion effects, these studies are not ideal for getting thorough estimates of the contribution of other factors influencing variation. Inversions are, without doubt, important for local adaptation, but they are also relatively easy to detect. Background effects, probably consisting of many small effect loci, can hardly be detected individually in association studies in general, and their contribution relative to inversion effects has not been quantified. However, well‐established quantitative genetics methods can be used to estimate their integrated contribution, summarised as V_A_, without aiming to identify single loci. Controlling for inversion effects by including them in our models allowed us to estimate V_A_, that is, variance due to polygenic background effects. It thus provides us with a unifying framework for evaluating the contribution of large effect loci while estimating polygenic effects at the same time.

In contrast to studies conducted in controlled laboratory environments, here we used field‐collected specimens. Controlled environments, for example, common garden experiments, have the benefit of avoiding confounding factors that can have a strong impact on plastic traits in natural conditions. Thus, they can confirm that inversions have a causal effect on certain traits and phenotypic divergence is not the result of plastic responses to different habitats. However, populations evolve in the presence of many environmental factors and their adaptation can only be fully understood in the environmental context and in presence of selection pressures that have shaped their evolution in the past. We found that habitat (substrate and wave exposure) as well as shore height explain some part of phenotypic variation, but their contribution is minor. Most of the phenotypic variation has a genetic basis (inversion polymorphisms as well as polygenic background effects). Interestingly, some inversions show very consistent effects whereas others seem to have site‐specific effects.

### Inversions contribute to ecotype divergence in replicated contact zones

Here, we show that inversion genotypes had significant effects on trait variation at three different sites under natural conditions. Some of the inversions showed consistent effects at all sites. For example, the inversion on LG 6 had a significant effect on shell length, which agrees with the QTL analysis that detected QTLs for size in this inversion region (Koch et al. 2021). The strongest effects in terms of significance and number of affected traits were shown by inversions on LG 12. LG 12 inversions have been less studied, but this LG has been found to be important for variation in several traits before (Koch et al. 2021). In addition, LGC 12.2 probably contains a sex‐determining locus (Koch et al. 2021; Hearn et al. 2022). *Littorina saxatilis* does not have heteromorphic sex chromosomes (García‐Souto et al. 2018) and LG 12 may represent a very early stage of sex chromosome evolution. It is expected that there should be reduced recombination between the sex‐determining locus and alleles under sexually antagonistic selection. We indeed found that some of the traits (height‐growth, width‐growth, aperture‐size) that were affected by LGC 12.2 showed sexual dimorphism (Fig. 2). However, the relationship between shell shape and sex‐specific fitness effects is still unknown.

Inversions that show very consistently extreme frequency differences for alternative arrangements between Crab and Wave habitats and accumulation (significantly increased density) of outliers SNPs (based on cline analysis (Westram et al. 2021) and between ecotype genetic differentiation (Morales et al. 2019)) are those on LG 6 and LG 14. However, in contrast to LG 6 we could only detect significant effects of LG 14 inversions at CZB (Fig. 2), although arrangement frequencies differed strongly between ecotypes at all sites (Fig. S2). This agrees with a previous study that could not detect QTLs within this region (Koch et al. 2021). Possibly, LG 14 influences traits under strong habitat‐specific selection that we did not measure (e.g., physiological traits).

We also found that some inversions showed site‐specific significant effects (e.g. LGC2.1 at CZD and LGC1.1 at CZA and CZB). Similar patterns were found previously. The inversion on LG 17 showed an accumulation of outlier SNPs (Westram et al. 2018; Morales et al. 2019) at a neighboring island and another study (Koch et al. 2021) using individuals from the same location confirmed the presence of QTLs for shell shape and aperture traits within this inversion. In contrast to this first study site, this inversion did not show an enrichment of Crab‐Wave outliers at any of the sites studied here (Westram et al. 2021) and we could not detect any phenotypic effects.

Overall, we see a mix of consistent as well as site‐specific effects. While the direction of effects was mostly the same, the effect size of inversions and their significance varied between sites despite similar variation in arrangement frequencies. How much an inversion contributes and how strongly it affects a phenotype probably depends on the genomic background, which may show site‐specific differentiation between ecotypes. Previous studies showed that sharing of Crab‐Wave outliers was increased in inversion regions (Morales et al. 2019; Westram et al. 2021) whereas overall outlier sharing was rather low despite the strong parallelism in phenotypic traits (Ravinet et al. 2016; Westram et al. 2016). While most inconsistencies in detecting the same outliers are probably explained by limited statistical power, low sharing could be partly caused by redundancy in the collinear genome where many loci contribute to phenotypic variation. Similar selection in the Crab and Wave habitats on different islands can lead to phenotypic convergence if different molecular pathways lead to similar phenotypes. This is especially the case if a trait is highly polygenic with some redundancy at the genetic level (Yeaman 2015; Barghi et al. 2019). Given that small‐additive‐effect loci strongly influenced phenotypic variation in most traits, it seems likely that the genetic underpinning of ecotype divergence may differ between sites and show unique patterns. However, occasional gene flow may have introduced variants with higher benefits, which may have increased in frequency. This can lead to a very similar distribution of inversion frequencies along habitat transitions with varying effect sizes on phenotypes. Alternatively, the adaptive content of inversions may differ between sites.

### Contributions of inversions and collinear regions

Although inversions had a strong effect, additive genetic effects of the collinear genomic background were substantial, and variance explained was of similar magnitude. This is consistent with a recent study using simulated data to show that even under conditions in which inversions are important they rarely explain more than 50–60% of the genetic variance (Schaal et al. 2022). In almost all traits, we found considerable contributions of inversions and additive genetic effects. The relative contribution varied among traits as well as among sites. At CZB, with a very narrow Crab habitat (Fig. 4) and a broad transition zone, the genetic differentiation between ecotypes was less pronounced compared to the other sites (Fig. S14). Here, the contribution of inversions to phenotypic variation was slightly higher and inversion effects showed a higher significance (Fig. 2a) compared to CZA where ecotypes showed the highest genetic differentiation in the genomic background (Fig. S14). This might be a confirmation that inversions, acting as large effect loci, can facilitate and accelerate local adaptation and development of distinct ecotypes when the risk of gene swamping is high. In contrast, we saw higher contributions of the genomic background at CZA where ecotypes showed the highest genetic differentiation.

### Environmental effects and sexual dimorphism

The overall pattern (Fig. 5) of phenotypic variance explained at each site suggests only minor contributions of habitat, sex, and shore height to total phenotypic variation. Overall, the phenotypic variation in traits considering the whole transect seems to be dominated by genetic effects, including additive genetic effects as well as inversion karyotypes. This agrees with previous studies demonstrating that ecotype divergence persists in the lab (Johannesson and Johannesson 1996) and association analyses supporting the role of inversion polymorphism for trait variation (Koch et al. 2021). However, this does not mean that other factors do not play an important role. Sex, as well as habitat, showed highly significant effects on several traits. Our analysis was done on the whole transect and therefore included phenotypic extremes. Comparing the inter‐ecotype differences to differences between females and males (Fig. 4b, Fig. S4), it is indeed obvious that ecotype differences are on a much larger scale. However, there are clear and consistent sex differences as well as effects of shore height and habitat. The effect of habitat was the strongest on shell shape and aperture‐related traits that had previously been shown to be plastic and able to respond to wave action and crab cues (Hollander and Butlin 2010).

Some traits (weight and height‐growth) were affected by shore height. This may result from selective effects or plastic responses, which are not necessarily mutually exclusive. A selective effect on size seems likely. Lower shore areas with occasionally strong wave action represent a high risk of dislodgement for larger snails since they cannot access small sheltered crevices (Raffaelli and Hughes 1978; Atkinson and Newbury 1984). Zones above a certain shore height might be less exposed and allow individuals to grow larger. We also found a significant effect on shell shape. Snails with a more Crab‐type shape have a lower ability to resist water flow (Le Pennec et al. 2017) and might have been removed from the low‐shore area. It was also shown that shape can plastically change in response to wave action (Hollander et al. 2006; Hollander and Butlin 2010), which may explain why high‐shore, probably less‐exposed individuals differ in shell shape from lower‐shore snails (see Fig. 4a, Figure S5).

We detected significant interactions between shore height and habitat for shape (height‐growth) and shell length meaning that the effect of different shore heights was not constant across the transect. The effect of shore height is likely to be most pronounced in the Wave habitat where there is a vertical gradient in wave exposure in contrast to the Crab habitat that is more sheltered overall. A general pattern (for all traits, not only those with significant shore effects) was that Wave snails at greater shore heights showed phenotypes more similar to the Crab ecotype (Fig. 4a; Fig. S5). However, interactive effects between shore and habitats should be interpreted with caution since variation in shore height was small and not equally distributed across the transect with the highest variation found in the Wave habitat.

We consistently found clear differences between sexes in size (weight and shell length), shell shape (height‐growth and width‐growth) as well as aperture size and aperture shape (Fig. 2 right panel; Fig. S4). The snails are direct developers where females carry developing embryos in a brood pouch. Larger size is likely to be beneficial for females since it enables them to produce considerably more offspring (Janson 1985). In contrast to size, sex‐specific selection on shell shape and aperture traits is not obvious. Potentially, certain adjustments in shell shape in females are required to accommodate a brood pouch.

### Relatedness across transects: reduced gene flow in habitat contact zone

Calculating median relatedness of individuals along the transect to surrounding ones showed that most of them were relatively closely related. The observation that first‐ and second‐degree relatives were found very close in space (within a few meters, Fig. S11) suggests that they have rather limited dispersal capacity (Janson 1983). This implies that subsequent generations stay in the same habitat and habitat‐specific selection acts on the genetic composition of the population over generations, which is crucial for evolving ecotypes on very small scales. At all sites, we observed a clear decrease in relatedness close to the habitat transition zone, independent of which distance we used for calculating median relationships (Fig. S12). Consistent with previous findings of cline shifts into the Wave habitat (Westram et al. 2021), this decline did not occur exactly at the habitat boundaries but was most pronounced around 10 m (dependent on site) into the wave habitat away from the transition. It seems likely that larger Crab individuals have a higher dispersal (see also Janson 1983) and are capable of moving into the wave habitat. The fact that relatedness dropped rapidly close to the habitat boundary strongly suggests that gene flow is reduced at this point. Homogeneous gene flow across the transect would result in a pattern of consistently close relationships to all surrounding individuals, that is, a more or less flat line in Fig. 6. The overall lower relationships to each other might suggest that the group of individuals close to the habitat transition was not maintained by interbreeding over several generations but are more likely the result of independent hybridization and migration events. This could indicate that there was no successful long‐term establishment of a population that could adapt to the specific conditions in the habitat transition. This pattern is in line with the cline analysis and shows that the population at this point is the result of combined migration and selection.

Divergent selection can lead to speciation (Hereford 2009; Nosil et al. 2009) if populations under different selection pressures evolve reproductive barriers to prevent maladaptive gene flow. Chromosomal inversions could contribute to speciation (Butlin 2005). They can lead to unbalanced gametes and non‐viable offspring, or they can facilitate speciation if alleles involved in local adaptation and other forms of reproductive isolation are linked (Felsenstein 1981). However, in *L. saxatilis* no evidence for effects of inversion karyotypes on embryo abortion rate could be detected (Johannesson et al. 2019). Some evidence for assortative mating (Perini et al. 2020) and ecotype‐specific habitat choice (in Spain, Rolán‐Alvarez et al. 1999) exists, and these barriers probably contribute to reproductive isolation, but their association with inversions is unknown. Although we found here some evidence that gene flow is reduced, there is still substantial hybridization between ecotypes.

### The contribution of large effect loci to phenotypic divergence under gene flow

This study contributes to our understanding of the genetics underlying phenotypic divergence in the presence of gene flow by quantifying the contribution of polymorphic chromosomal inversions acting as large effect loci, in relation to the remaining genomic background and plastic responses to different environments. It is known that certain genetic architectures make local adaptation more resistant against the homogenizing effects of gene flow (Bürger and Akerman 2011; Yeaman and Whitlock 2011; Tigano and Friesen 2016). These include large effect loci, pleiotropic loci that affect multiple traits under divergent selection, and a clustering of adaptive loci in the genome or alternatively, regions without recombination, i.e. where mixing of the differently adapted genomes is prevented. Our observation that the same inversions influenced multiple traits fits well with predictions that inversions can act as supergenes by containing sets of locally adaptive alleles (Faria et al. 2019b; Tigano and Friesen 2016; Wellenreuther et al. 2019). The lack of recombination in individuals that are heterozygous for alternative arrangements can preserve beneficial combinations even in presence of extensive gene flow and allows co‐segregation of adaptive variation within species. However, we cannot exclude alternative mechanisms, like pleiotropic alleles inside the inversions or the effects of the inversion events themselves that may have changed the expression of multiple genes. Regardless of the exact mechanism, we could confirm that inversions act as large effect loci and have shown that large proportions of the phenotypic variation in a contact zone are indeed explained by inversion polymorphisms. Most inversion effects were very consistent across sites, which provides additional support for their impacts on traits under divergent selection. While we found clear evidence for inversions being involved in parallel ecotype evolution, the overall picture is more complex. How much inversions contribute differs between sites and the proportion explained by small effect loci in the genomic background is substantial. If gene flow is smaller and overall genetic differentiation higher, the contribution of small effect loci is likely to become larger, which results in a lower relative contribution of large effect loci. It should be kept in mind that the importance of large effect loci can only be fully understood with some knowledge of the genomic background contribution. Furthermore, environmental effects can have substantial impacts as well. Considering all these contributing factors jointly will give us a more complete picture of the genetic architecture of local adaptation.

## AUTHOR CONTRIBUTIONS

M.R., R.K.B., K.J., and A.M.W. designed the project and collected the data. E.L.K. led the data analysis and wrote the manuscript with input from all authors.

## CONFLICT OF INTEREST

The authors declare no conflict of interest.

## DATA ARCHIVING

Data is available from Dryad (https://doi.org/10.5061/dryad.m905qfv4b).

Associate Editor: E. Sotka

Handling Editor: T. Chapman

## Supporting information

Supplementary Information

Supplementary Information
