# Supplementary material for: Genetic architecture of repeated phenotypic divergence in Littorina saxatilis ecotype evolution
Source: Evolution. 2022 Sep 1;76(10):2332–46. doi: 10.1111/evo.14602 (PMC9826283; doi:10.1111/evo.14602)
Supplement: Supplementary file 1 — Supplementary Information [file EVO-76-2332-s002.pdf]

Supporting Information

Figure S1: PCA on markers within inverted regions on LG 12.

Figure S2: Distribution of inversion genotypes along the transect.

Figure S3: Trait values along the transect coloured by habitat.

Figure S4: Differences between females and males of both ecotypes in different traits.

Figure S5: Trait values along the transect coloured by shore height.

Figure S6: Variances explained by combined effects.

Figure S7: Significance of inversion effects at three locations when "Distance from centre" is excluded.

Figure S8: Variances of inversion genotypes at each site.

Figure S9: Variances of non-standardised traits at each site.

Figure S10: Effect sizes of inversions for all traits at the three sites.

Figure S11: Pairwise relationships between all individuals from the same site and the corresponding distance between individuals along the transect.

Figure S12: Median relatedness of individuals to others within varying distances along the transect.

Figure S13: Variance explained by different linkage groups in relation to their size.

Figure S14: Average F_{ST} between ecotypes from the same site excluding SNPs from inversion regions.

Table S1: Results of the statistical analysis.

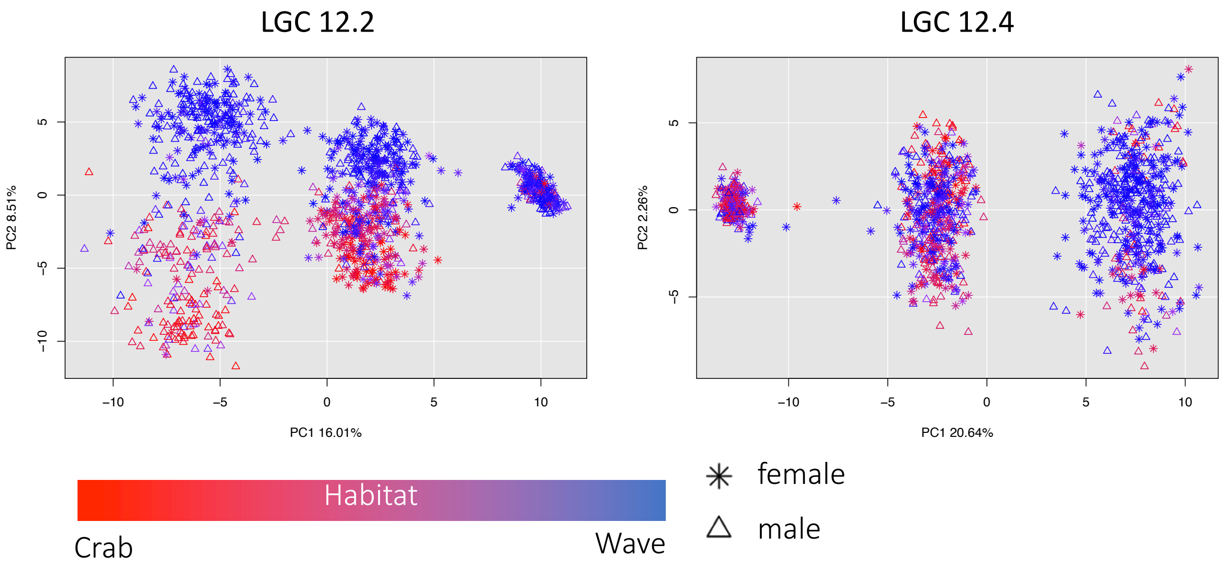

Figure S1: PCA based on markers within putatively inverted region on LG 12. Samples are coloured according to the habitat PC (Crab habitat = red, Wave habitat = blue)

CZA

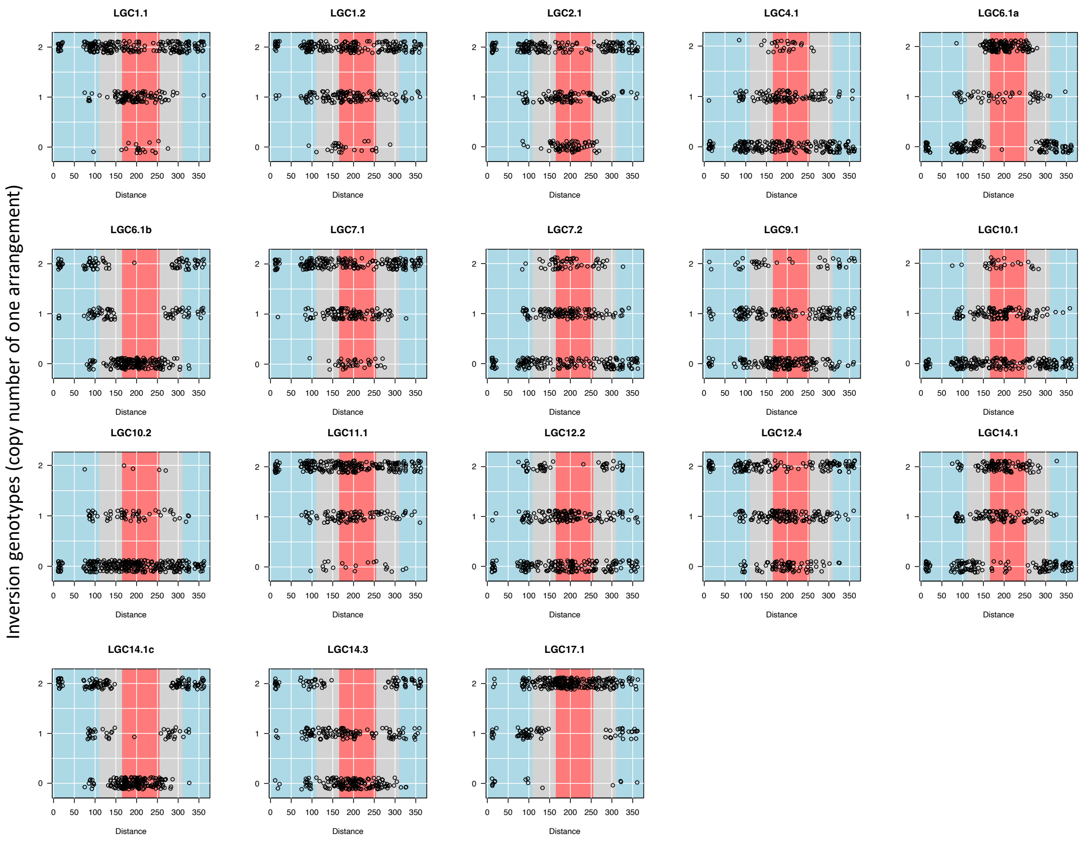
LGC1.1
LGC1.2
LGC2.1
LGC4.1
LGC6.1a
LGC6.1b
LGC7.1
LGC7.2
LGC9.1
LGC10.1
LGC10.2
LGC11.1
LGC12.2
LGC12.4
LGC14.1
LGC14.1c
LGC14.3
LGC17.1
Distance
Inversion genotypes (copy number of one arrangement)

CZB

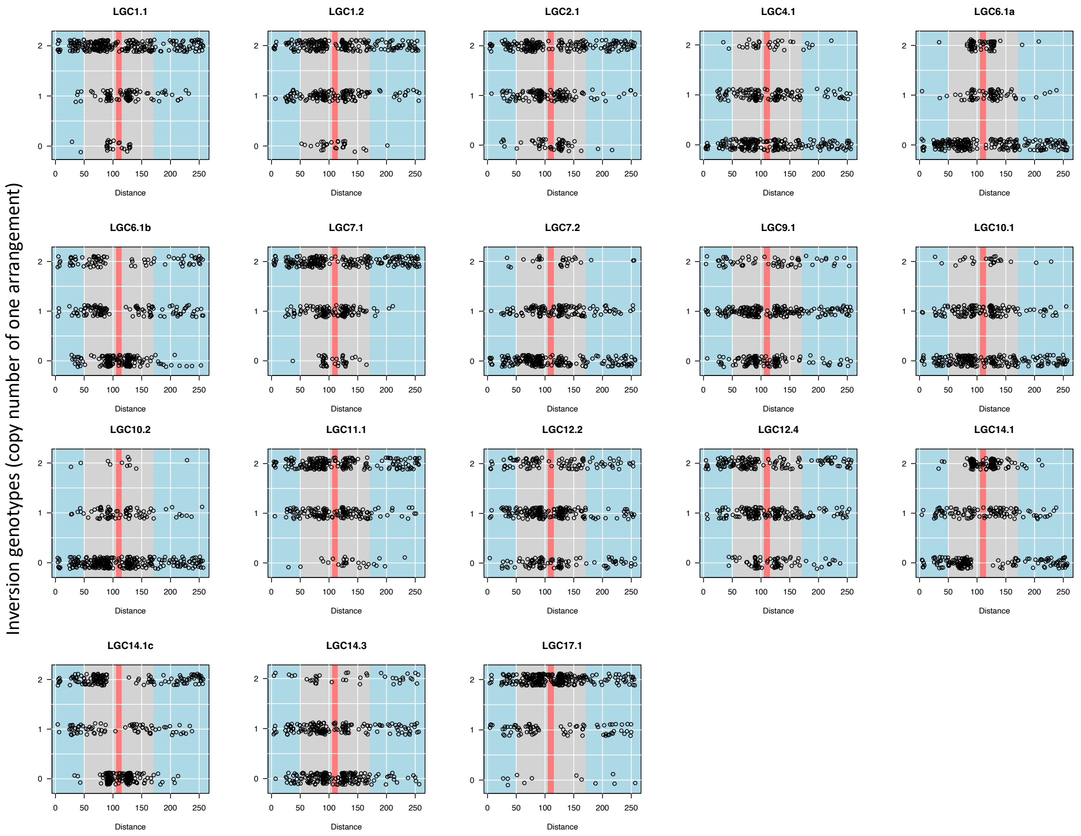
LGC1.1
LGC1.2
LGC2.1
LGC4.1
LGC6.1a
LGC6.1b
LGC7.1
LGC7.2
LGC9.1
LGC10.1
LGC10.2
LGC11.1
LGC12.2
LGC12.4
LGC14.1
LGC14.1c
LGC14.3
LGC17.1
Distance
Inversion genotypes (copy number of one arrangement)

CZD

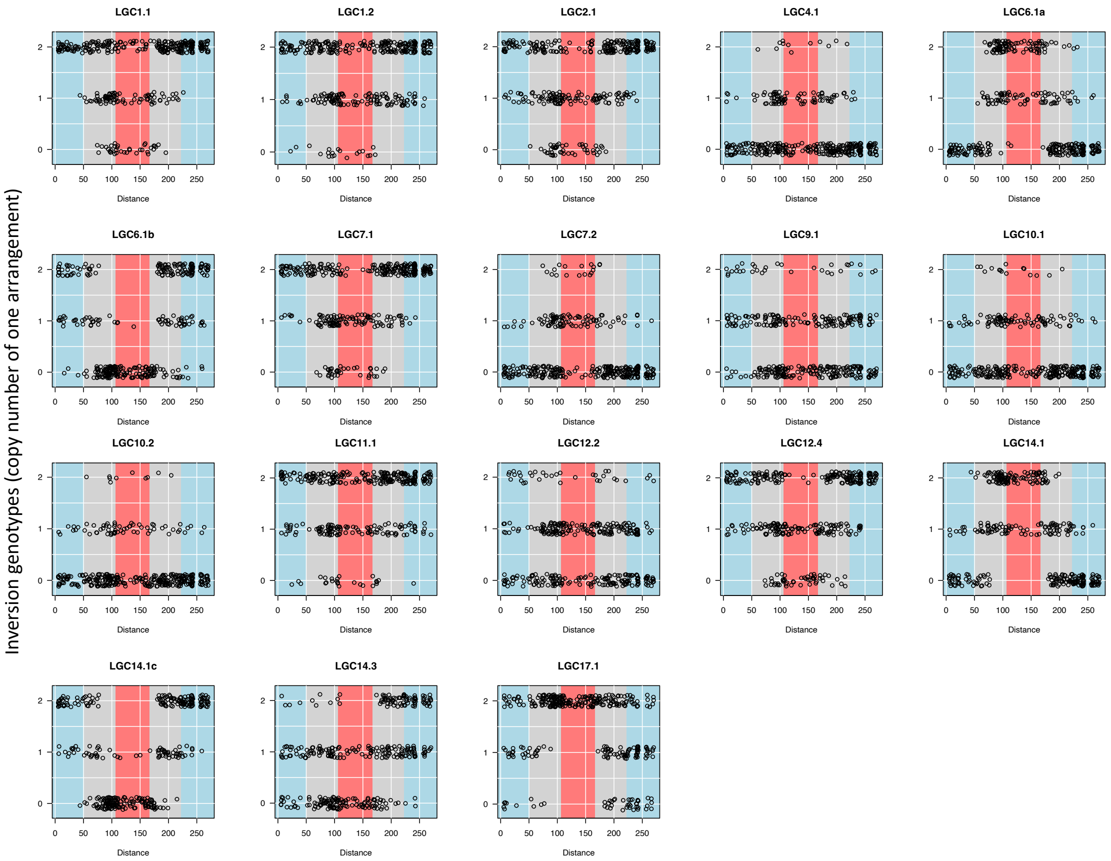

Figure S2: Distribution of inversion genotypes along the transect (0 and 2: homozygous, 1: heterozygous). Background colours refer to the Wave (blue) and Crab (red) habitat, grey indicates the transition zone.

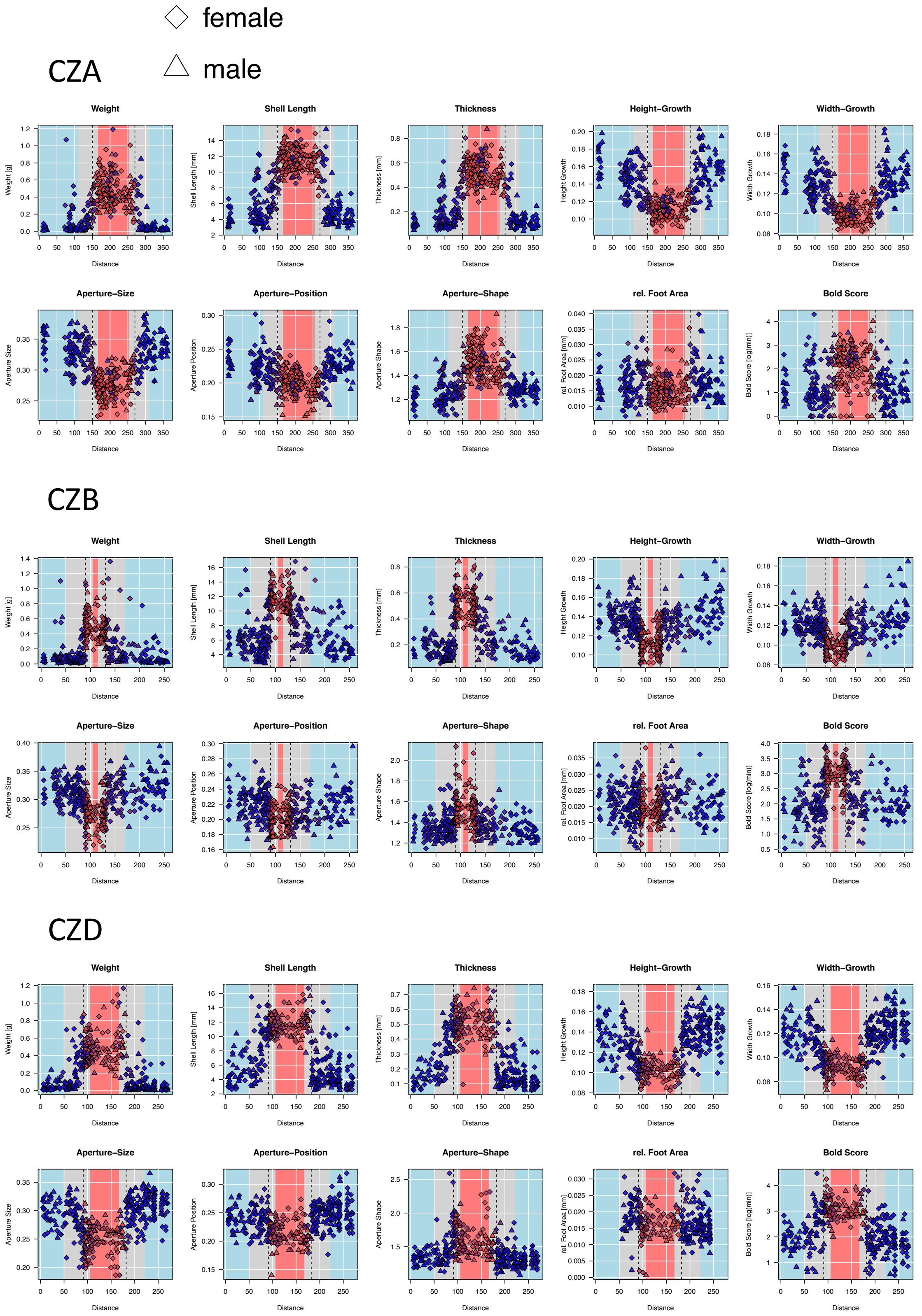

Figure S3: Trait values along the transect coloured by habitat PC (red = Crab habitat, blue = Wave habitat). Background colours refer to the areas defined as Crab (red) and Wave (blue) habitat and the transition zone (grey).

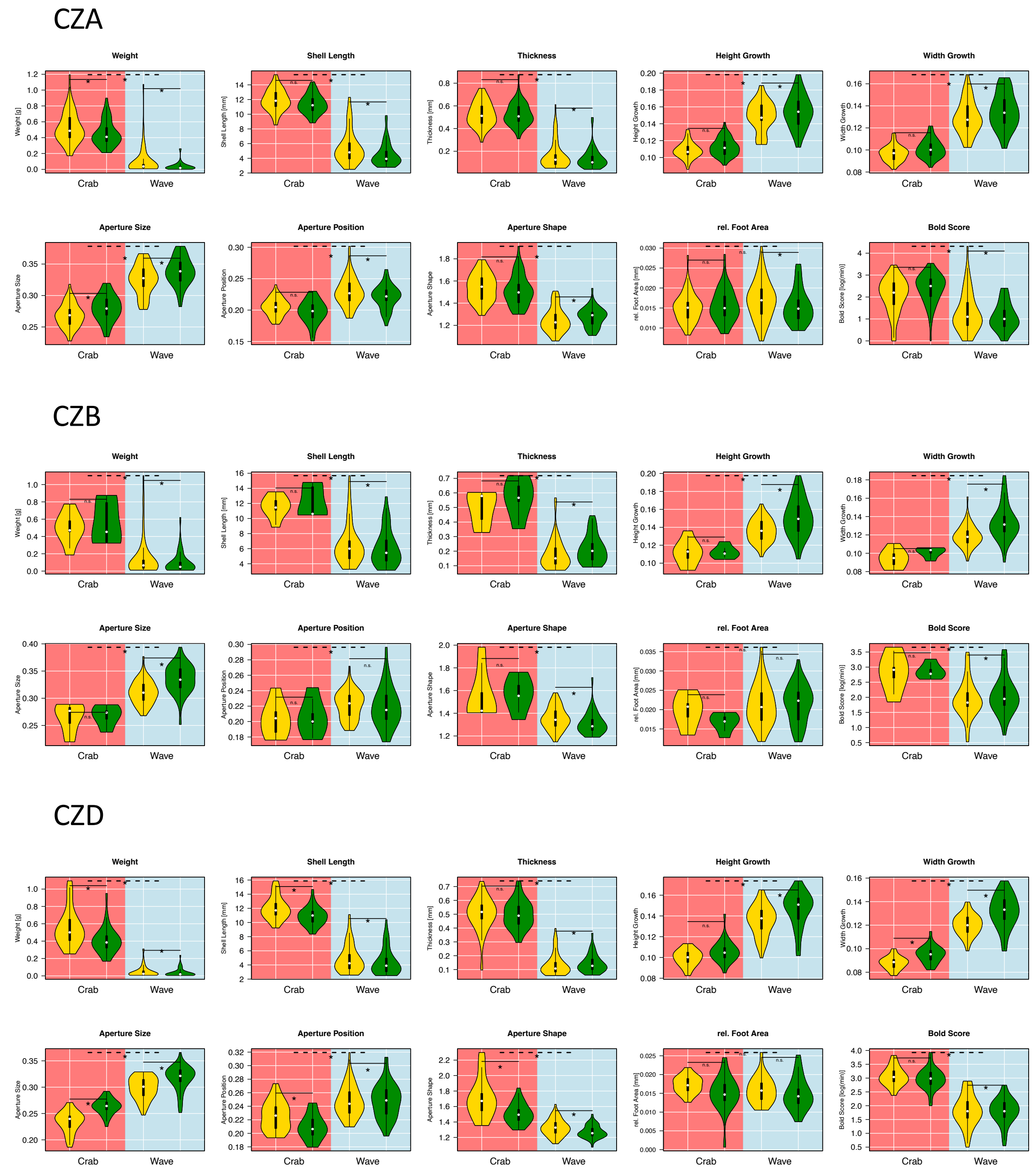

Figure S4: Differences between females (yellow) and males (green) of both ecotypes in different traits at CZA, CZB, and CZD. Background colours indicate ecotypes: red = Crab, blue= Wave. Most traits showed significant differences between snails sampled in the Crab and Wave habitat as well as between sexes.

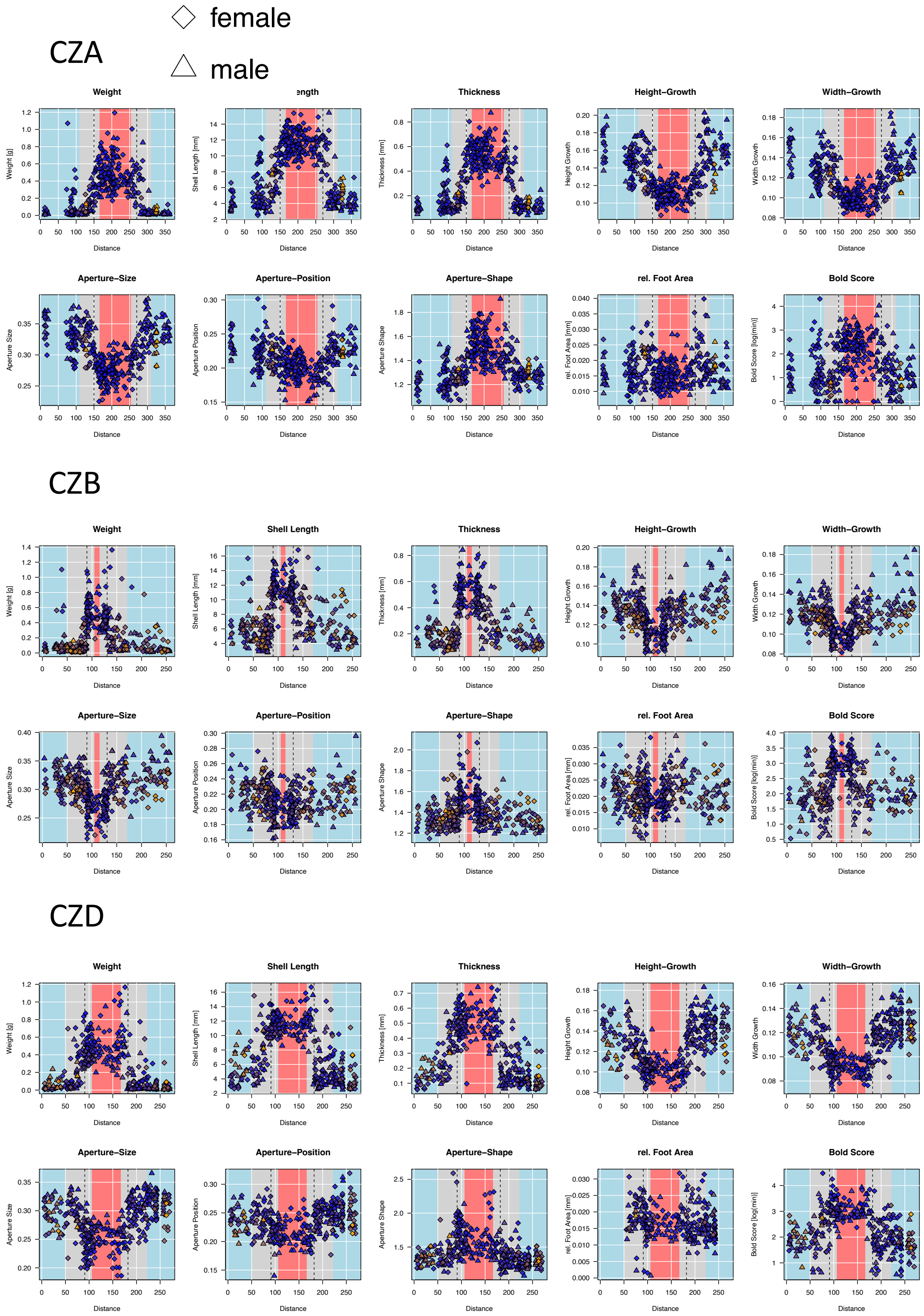

Figure S5: Trait values along the transect coloured by shore height (yellow = upper shore, blue = lower shore).

CZA

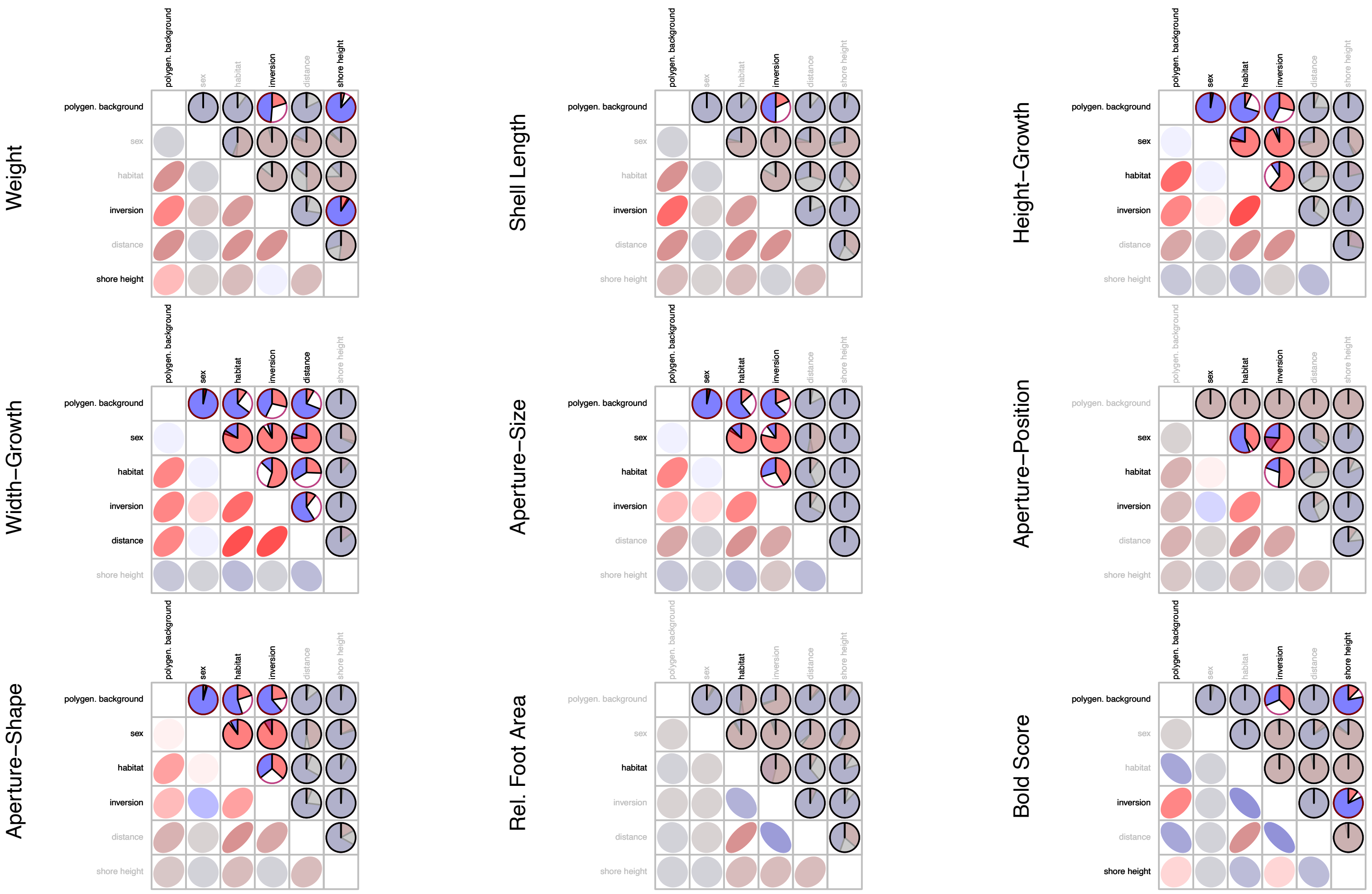
Weight
Shell Length
Height–Growth
Width–Growth
Aperture–Size
Aperture–Position
Aperture–Shape
Rel. Foot Area
Bold Score
polygen. background
sex
habitat
inversion
distance
shore height

CZB

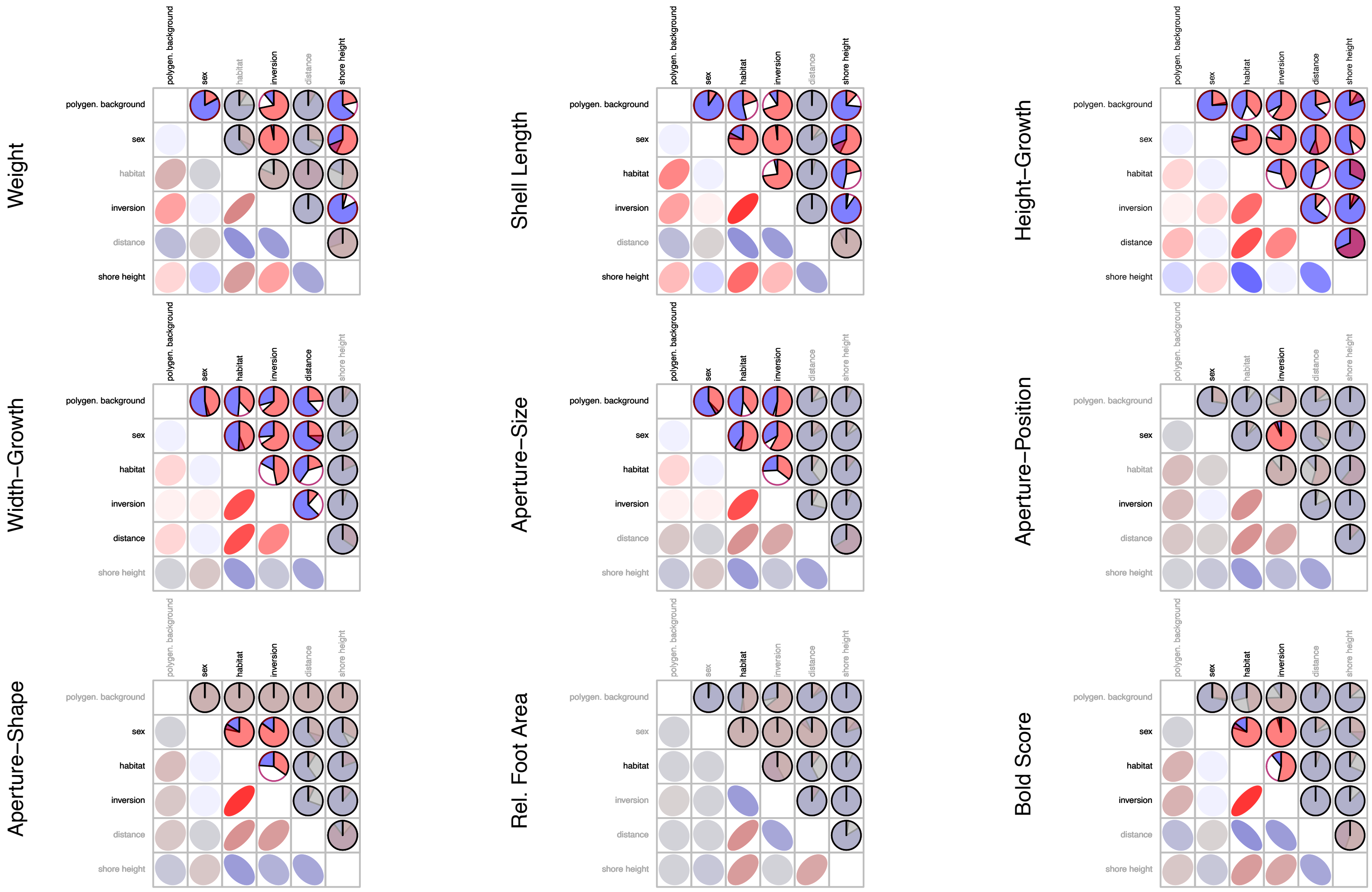

Weight
Shell Length
Height–Growth
Width–Growth
Aperture–Size
Aperture–Position
Aperture–Shape
Rel. Foot Area
Bold Score
polygen. background
sex
habitat
inversion
distance
shore height

CZD

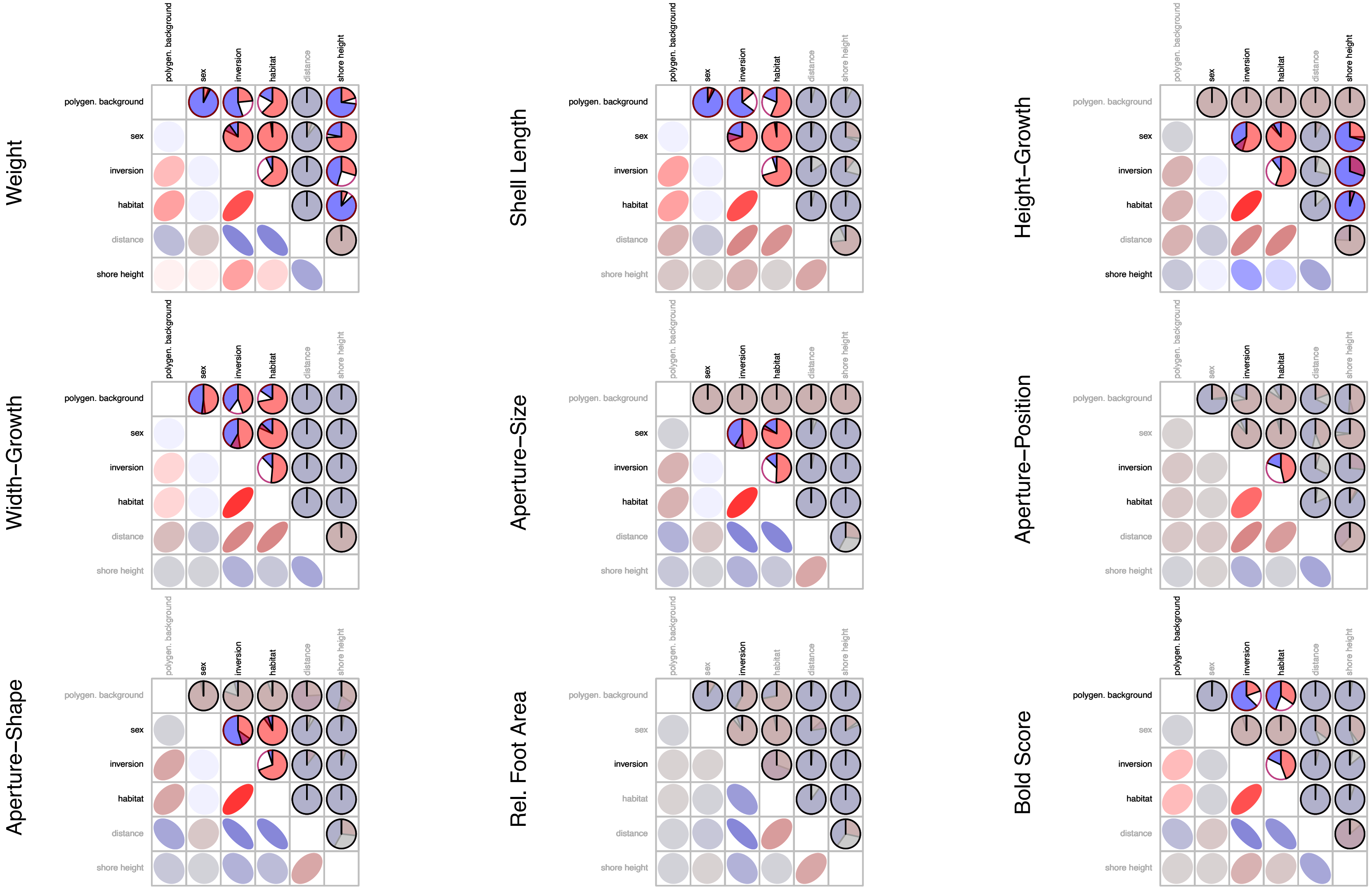
Weight
Shell Length
Height–Growth
Width–Growth
Aperture–Size
Aperture–Position
Aperture–Shape
Rel. Foot Area
Bold Score
polygen. background
sex
inversion
habitat
distance
shore height

Figure S6: Variances explained by combined effects. Variances explained by fixed effects were calculated based on de Villemereuill et al. 2018. If there is collinearity between variables, the variance explained by their combined effect is higher than the sum of variances explained by single factors. The difference corresponds to twice the covariance between predicted values. Lower triangle: Correlation between predicted values. Upper triangle: Circles represent the total amount of variance that is explained by a pair of predictors. Blue = variance uniquely explained by factor of the row; red = variance uniquely explained by factor of respective column; white = positive covariance; purple = negative covariance. Negative covariance indicates counteracting effects of the two predictors, for example shore height and inversion effects at CZD or shore height and habitat at CZD. Snails collected at higher shore heights show indeed phenotypes that are different from the phenotypes expected based on inversion genotypes and habitat, see Figure S5 or Figure 4A in the main manuscript. Non-significant factors are shown in grey.

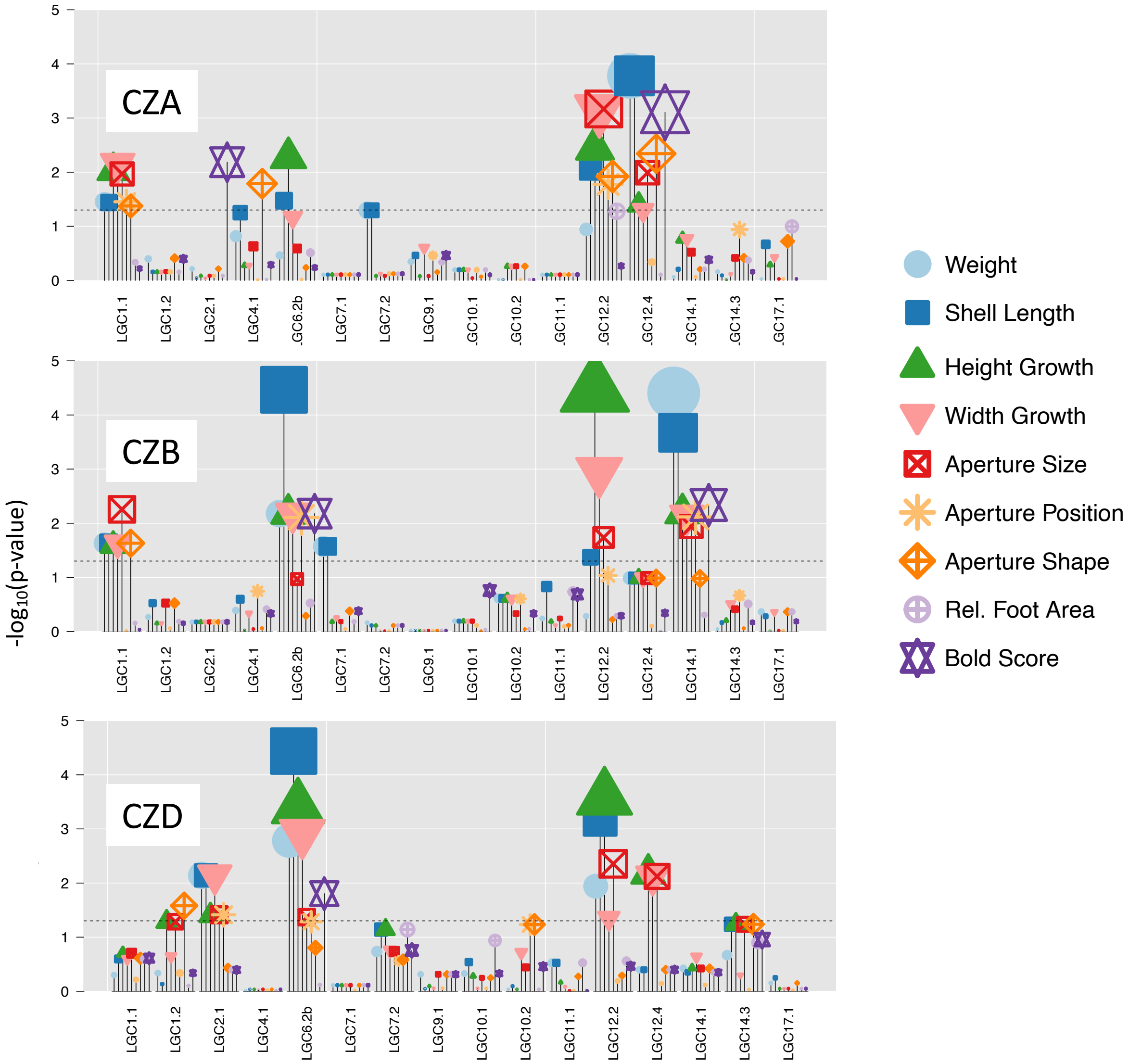

Figure S7: Significance of inversion effects at three locations when “Distance from centre” is excluded. Shown are -$\log_{10}$ of p-values on the y-axis. Significance was assessed by using conditional Wald-tests. p-values were adjusted for testing multiple traits using the false discovery rate (FDR). The dashed line indicates the significance threshold (FDR = 0.05). Effects of inversions are shown on the left, additional effects on the right.

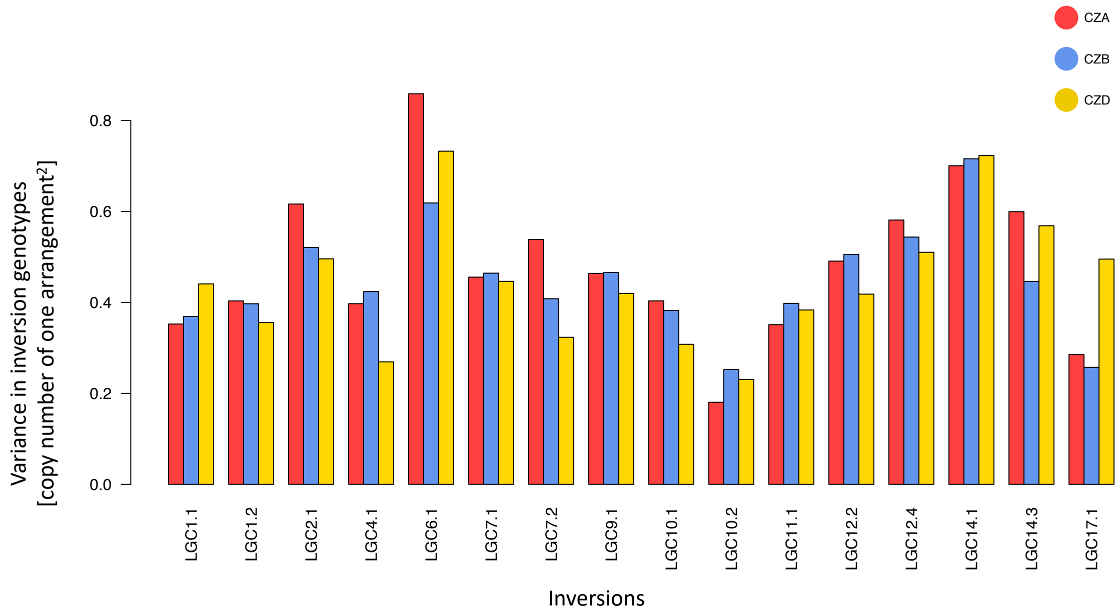

Figure S8: Variances of the frequency of one of the inversion arrangements (i.e., variance of the inversion genotypes of all individuals coded as 0 and 2 for homozygous and 1 for heterozygous) for each site.

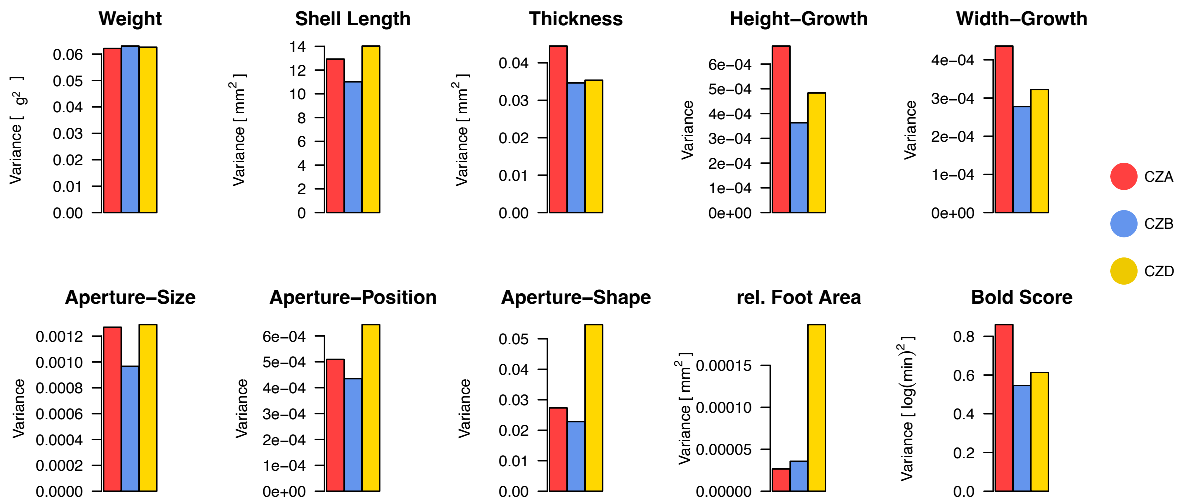

Figure S9: Variances of non-standardised phenotypic values of each site.

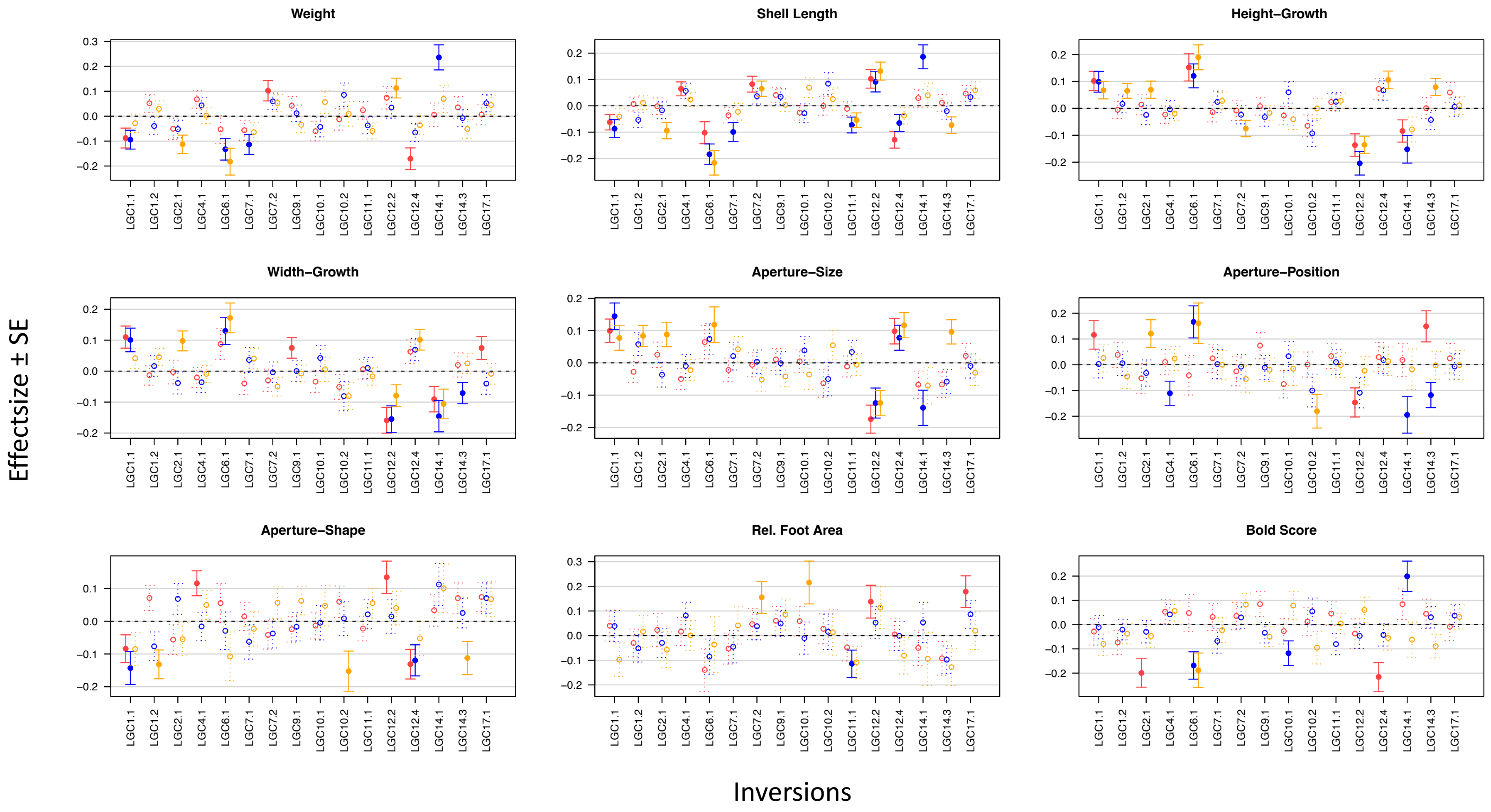

Figure S10: Estimated inversion effects (± Standard Error). Traits and inversion genotypes were standardized and analysed for each site separately. Dashed lines and open circles represent non-significant effects (based on P-values that are not corrected for testing multiple traits).

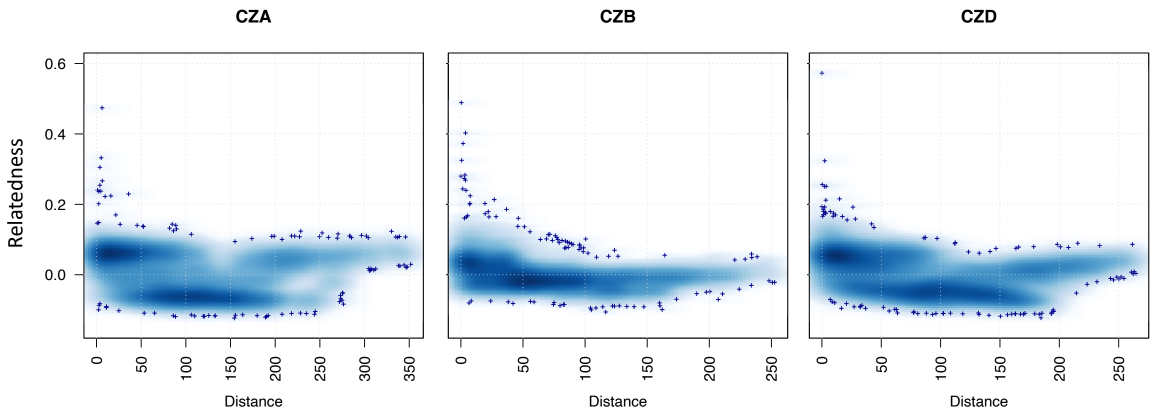

Figure S11: Pairwise relationships between all individuals from the same site and the corresponding distance (in meters) between individuals along the transect. Colour density corresponds to density of data points; outliers are shown as single points. Closely related individuals (first- and second-degree relatives) are also close in space and usually less than ten meters apart. Only SNPs outside inversion regions were used to calculate genomic relationships.

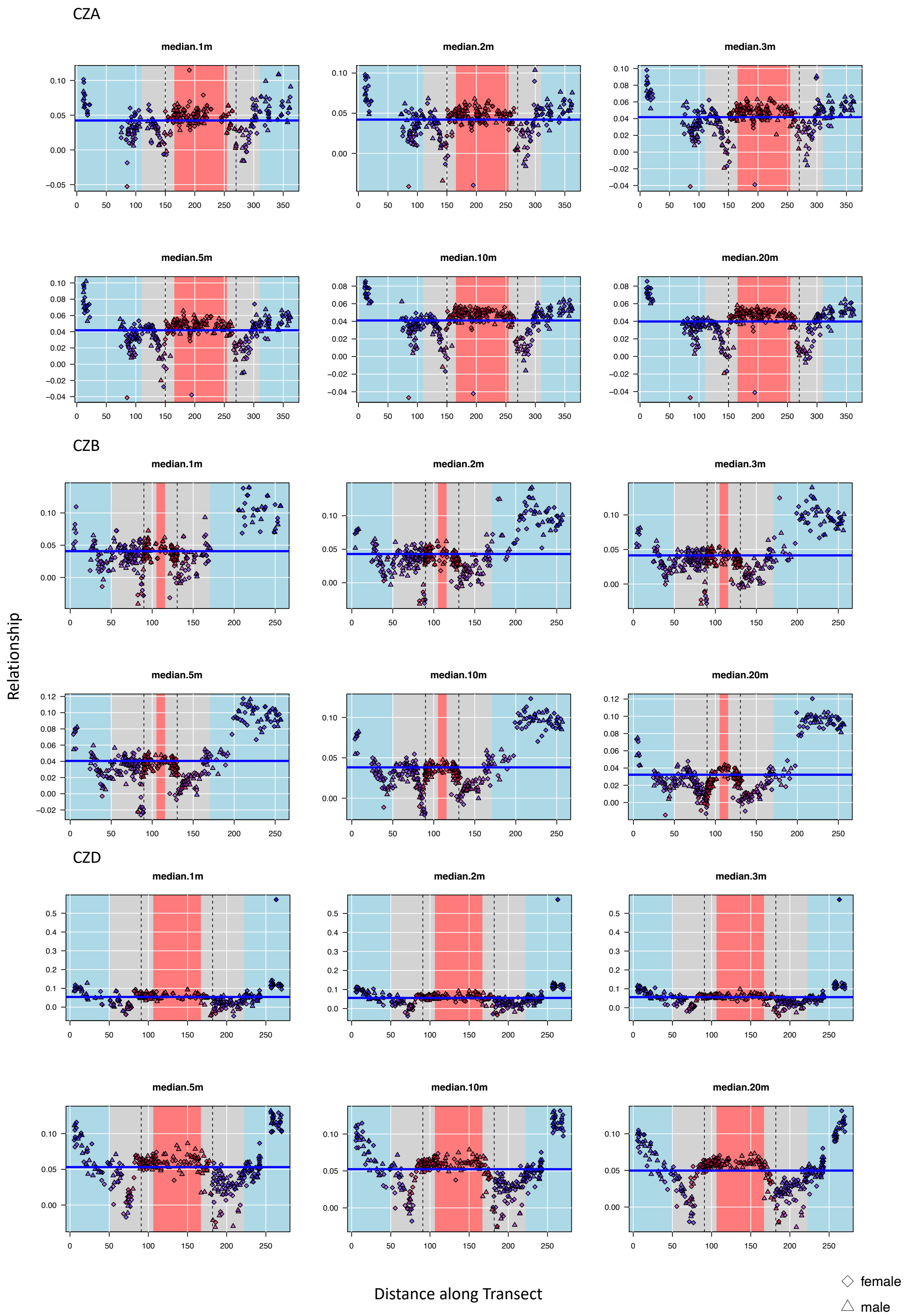

Figure S12: Median relatedness of individuals to others within varying distances along the transect. Colours refer to hybrid indices (red = pure Crab, blue = pure Wave). The vertical blue lines indicate the boundaries of the “pure” Wave habitat, the red lines of the Crab

habitat. Dashed lines indicate the habitat transition. The blue horizontal line shows the average relatedness within the respective distance.

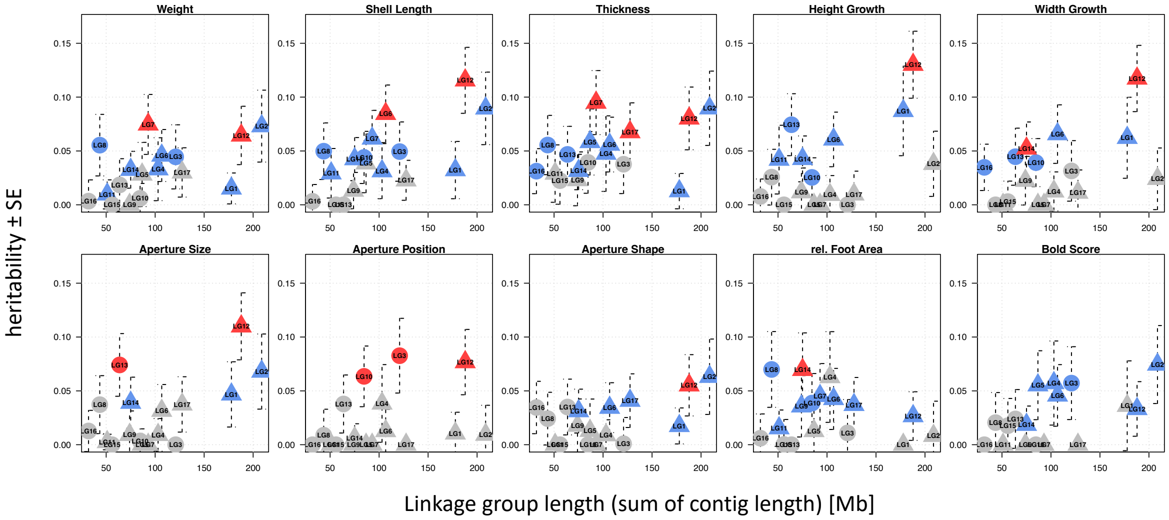

Figure S13: Variance explained by different linkage groups (LG) ± standard error (SE) relative to sum of lengths of contigs that are assigned to each LG (proportional to chromosome length). If a trait is completely polygenic and loci are evenly distributed across chromosomes, a positive correlation between linkage group length and variance explained is expected and often several LGs contribute significantly. Deviations from polygenicity can be caused by large effect loci or clustering of loci. LGs explaining significant amounts of phenotypic variance are shown in blue; those explaining more phenotypic variance than expected based on their length in red. LGs harbouring inversions are indicated by triangles. For more details, see Koch et al. (2021), a previous study where this method was applied to laboratory-reared families of *L. saxatilis*. Data of all three sites were combined and site included as a fixed factor the models.

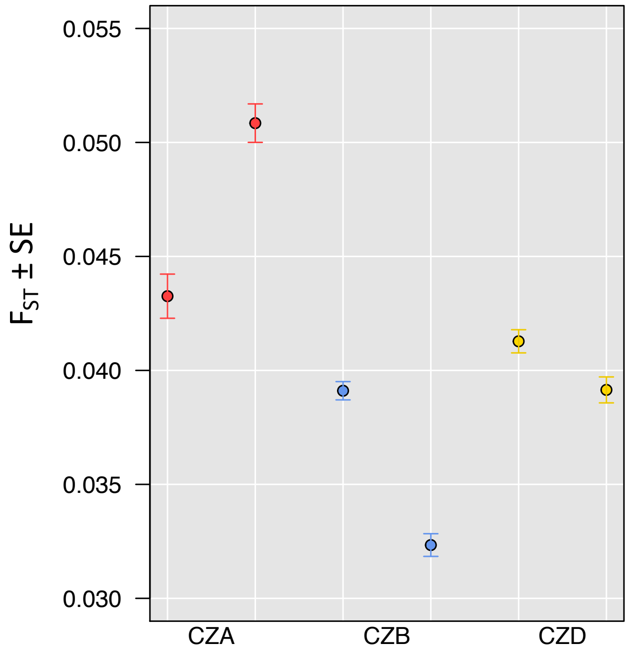

Figure S14: Average F_{ST} between ecotypes from the same site excluding SNPs from inversion regions. F_{ST} between Crab and the Wave populations on the left side of the bay is shown on the left, F_{ST} to the right Wave populations on the right side.
